# Simultaneously Controlling Inflammation and Infection by Smart Nanomedicine Responding to the Inflammatory Microenvironment

**DOI:** 10.1002/advs.202403934

**Published:** 2024-09-03

**Authors:** Xinjing Lv, Jie Min, Jie Huang, Hairong Wang, Song Wei, Chenxiao Huang, Jianfeng Dai, Zhengrong Chen, Huiting Zhou, Yunyun Xu, He Zhao, Zhuang Liu, Jian Wang

**Affiliations:** ^1^ Children's Hospital of Soochow University Pediatric Research Institute of Soochow University Suzhou Jiangsu 215123 China; ^2^ Institutes of Biology and Medical Sciences Jiangsu Key Laboratory of Infection and Immunity Soochow University Suzhou Jiangsu 215123 China; ^3^ Institute of Functional Nano & Soft Materials (FUNSOM), Jiangsu Key Laboratory for Carbon‐Based Functional Materials & Devices Soochow University Suzhou Jiangsu 215123 China

**Keywords:** anti‐infection and anti‐inflammation treatment, bioluminescence resonance energy transfer, inflammatory microenvironment, myeloperoxidase, neutrophils

## Abstract

The overactivated immune cells in the infectious lesion may lead to irreversible organ damages under severe infections. However, clinically used immunosuppressive anti‐inflammatory drugs will usually disturb immune homeostasis and conversely increase the risk of infections. Regulating the balance between anti‐inflammation and anti‐infection is thus critical in treating certain infectious diseases. Herein, considering that hydrogen peroxide (H_2_O_2_), myeloperoxidase (MPO), and neutrophils are upregulated in the inflammatory microenvironment and closely related to the severity of appendectomy patients, an inflammatory‐microenvironment‐responsive nanomedicine is designed by using poly(lactic‐co‐glycolic) acid (PLGA) nanoparticles to load chlorine E6 (Ce6), a photosensitizer, and luminal (Lum), a chemiluminescent agent. The obtained Lum/Ce6@PLGA nanoparticles, being non‐toxic within normal physiological environment, can generate cytotoxic single oxygen via bioluminescence resonance energy transfer (BRET) in the inflammatory microenvironment with upregulated H_2_O_2_ and MPO, simultaneously killing pathogens and excessive inflammatory immune cells in the lesion, without disturbing immune homeostasis. As evidenced in various clinically relevant bacterial infection models and virus‐induced pneumonia, Lum/Ce6@PLGA nanoparticles appeared to be rather effective in controlling both infection and inflammation, resulting in significantly improved animal survival. Therefore, the BRET‐based nanoparticles by simultaneously controlling infections and inflammation may be promising nano‐therapeutics for treatment of severe infectious diseases.

## Graphical abstract

In the inflammatory microenvironment, Lum/Ce6@PLGA nanoparticles can be oxidized by HClO, the MPO‐catalyzed product, generating light to stimulate Ce6 for the further production of ^1^O_2_, which can kill pathogens and induce the apoptosis of the excessive neutrophiles in the inflammatory lesion for alleviated tissue damage and improved animal survival in various peritonitis and pneumonia models.

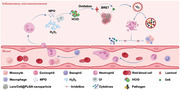

## 1 Introduction

Infectious diseases, including pulmonary infections and intestinal infections, have always been a threat to public health.^[^ ^1^, ^2^ ^]^ Antibiotics are usually used to control bacterial infections in clinic. However, it has been found that antibiotics can induce imbalance of gut microbiota and thus certain adverse reactions.^[^ ^3^ ^]^ Furthermore, the abuse of antibiotics would lead to the emergence and spread of multidrug‐resistant bacteria.^[^ ^4^, ^5^ ^]^ Meanwhile, it is very important to regulate the balance between anti‐inflammation and anti‐infection treatment in some inflammatory diseases such as lupus erythematosus.^[^ ^6^, ^7^, ^8^ ^]^ The patients with these diseases would usually encounter infections as a result of long‐term use of anti‐inflammatory drugs that have off‐targeting effects and may induce systemic immunosuppression.^[^ ^6^, ^7^, ^8^ ^]^ Therefore, there is an urgent need for the development of new therapeutic strategies that could simultaneously achieve anti‐inflammatory and anti‐infection therapies without impairment of immune homeostasis in clinic.

It has been well recognized that in tissues or organs invaded by bacteria or viruses, there usually exist unique inflammatory microenvironments, in which pathogens may grow to change the homeostasis in the local environment and further form many new characteristics such as reduced pH and upregulated reactive oxygen species (ROS).^[^ ^9^, ^10^ ^]^ In the meanwhile, various pro‐inflammatory cytokines together with different types of infiltrated immune cells including monocytes, macrophages and neutrophils, would be found at increased levels in the inflammatory lesion. The inflammatory microenvironment with the above characteristics usually could lead to increased inflammatory injury.^[^ ^11^, ^12^ ^]^ In particular, it is well known that neutrophils play an important role in anti‐infectious immunity and inflammatory responses.^[^ ^13^, ^14^ ^]^ Upon infection, neutrophils would be recruited to the infective site and release myeloperoxidase (MPO) and elastase.^[^ ^15^, ^16^ ^]^ The released MPO could trigger the generation of hypochlorous acid (HClO) from endogenous chloride ions and upregulated hydrogen peroxide (H_2_O_2_) to kill bacteria.^[^ ^17^, ^18^ ^]^ However, the excessive recruitment, activation, and prolonged survival of neutrophils can lead to the occurrence of chronic diseases and irreversible organ damages in autoimmune and inflammatory diseases, such as acute lung injury and sepsis.^[^ ^19^, ^20^ ^]^Therefore, it is vital to develop a new treatment approach that can not only effectively kill the pathogens, but also regulate the lesion microenvironment by reducing the excessive infiltration of inflammatory immune cells such as neutrophils, in order to achieve a synergy of anti‐infection and anti‐inflammatory therapies.

**Figure 1:**
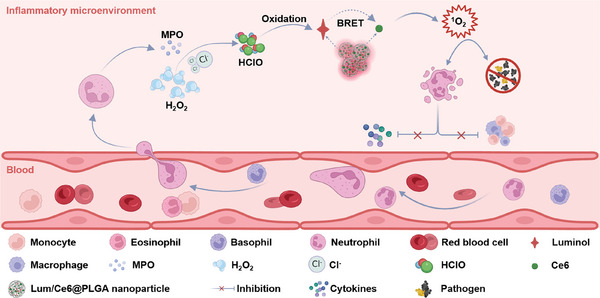
Schematic illustration of the Lum/Ce6@PLGA nanoparticles to respond inflammatory microenvironment for improved anti‐infection and anti‐inflammation therapy.

In recent years, various types of nanoparticles have been used in detection and treatment for inflammatory diseases.^[^ ^21^, ^22^, ^23^ ^]^ Recent studies have shown that MPO is wildly expressed in the inflammatory sites.^[^ ^24^, ^25^, ^26^ ^]^ Therein, MPO‐responsive biomaterials via bioluminescence resonance energy transfer (BRET) can be used for inflammation imaging or tumor therapy through BRET‐mediated photodynamic therapy.^[^ ^24^, ^25^, ^26^ ^]^ However, the use of BRET‐based nanomedicine to treat infective diseases has not yet been investigated and the unique advantages of such applications remain to be demonstrated. In this work, we first analyzed the data from different patients to confirm the characteristics of the inflammatory microenvironment, including large amounts of infiltrated neutrophils, as well as upregulated H_2_O_2_ and MPO levels. It was further found that the percentage of neutrophils in the inflammatory microenvironment was gradually increased with the disease severity, and positively correlated with the length of hospital stay. Therefore, we designed a nanomedicine system based on BRET that could respond to and further regulate the characteristics of the inflammatory microenvironment for improved anti‐infection and anti‐inflammatory therapy (**Figure** **1**). In our design, we constructed poly (lactic‐co‐glycolic acid) (PLGA) nanoparticles loaded with a photosensitizers chlorin e6 (Ce6) and a chemiluminescence donor luminol (Lum). In the inflammatory microenvironment, luminol in our synthesized Lum/Ce6@PLGA nanoparticles could be oxidized by HClO, the MPO‐catalyzed product (H_2_O_2_ oxidization of chloride ion), generating light to stimulate Ce6 for the further production of ^1^O_2_, which could subsequently kill pathogens and induce the apoptosis of the excessive neutrophiles in the inflammatory lesion. As the results, Lum/Ce6@PLGA nanoparticles by using topical injection or inhalation could not only eliminate bacterial infection, but also reduce inflammation, resulting in greatly alleviated tissue damage and improved animal survival in various peritonitis and pneumonia models. Therefore, such BRET‐based smart nanoparticles, Lum/Ce6@PLGA, could act as a unique type of nano‐therapeutics to provide rather effective protection against infectious and inflammatory diseases.

## 2 Results

### 2.1 The Characteristics of Inflammatory Microenvironment for Patients in Clinic

**Figure 2:**
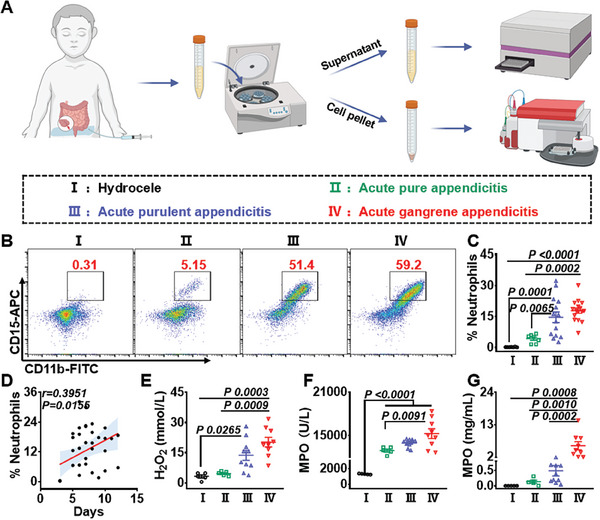
The characteristics of microenvironment in the patients with infectious acute appendicitis in clinic. A) A scheme for the analysis of effusion in clinical cases. B,C) Representative flow cytometry graphs (B) and statistical data (C) of the proportions of neutrophils in different groups as indicated. D) The correlation between the date of hospital admission and the proportions of neutrophils in effusion of different patients. E) The concentrations of H_2_O_2_ in the effusion of different patients. (F,G) The activities (F) and concentrations (G) of MPO in different groups as indicated. Data are presented as mean ± SEM. Statistical significance was calculated by one‐way ANOVA with Tukey's post hoc test or Pearson correlation.

The inflammatory microenvironment is formed by the interactions between pathogens and immune cells.^[^ ^27^, ^28^ ^]^ Neutrophils, the first line of innate immune cells in responsive to infections, can migrate to the site of infection and kill bacteria by various meanings such as phagocytosis, degranulation, and the release of neutrophil extracellular traps.^[^ ^29^, ^30^ ^]^ Therefore, the concentrations of MPO released by neutrophils and H_2_O_2_ in the inflammatory microenvironment may be important indicators to assess the seriousness of inflammation.^[^ ^31^, ^32^ ^]^ In our work, the proportion of neutrophils and the concentrations of H_2_O_2_ and MPO were thus detected in the intrathecal effusion of children with non‐inflammatory hydrocele, as well as the peritoneal fluid of children with infectious acute appendicitis (**Figure** **2**A). It was found that the proportion of neutrophils in the peritoneal fluid collected from patients with acute appendicitis was significantly higher compared that collected from children with hydrocele (Figure 2B,C). Furthermore, the percentage of neutrophils was gradually increased with the increased severity of acute appendicitis (Figure 2B,C). Notably, a positive correlation was found between the percentage of neutrophils in the infectious site and the hospital stay as calculated by Peason correlation (r = 0.3951, P = 0.0155) (Figure 2D), indicating that retention of neutrophils in the infection site could impede the recovery process in the patients. Meanwhile, the levels of H_2_O_2_, the activity, and concentration of MPO in the effusion increased progressively with disease progression (Figure 2E‐G). The above results demonstrated that the inflammatory microenvironment would be formed in infectious acute appendicitis. The number of neutrophils and the levels of H_2_O_2_ and MPO were obviously elevated in the microenvironment of acute appendicitis. Furthermore, the increased neutrophils could imply the aggravated severity of diseases and delayed patient recovery, demonstrating the importance of inflammatory microenvironment for the progress of patients with critical infections.

### 2.2 Synthesis, Characterization, and In Vitro Functions of Lum/Ce6@PLGA Nanoparticles

**Figure 3:**
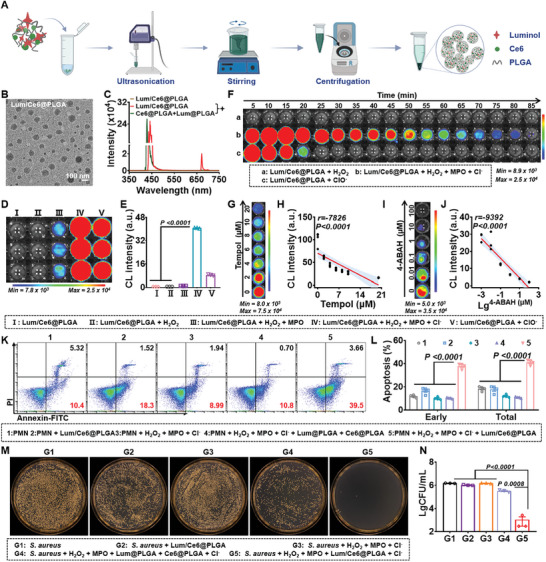
Characterization and in vitro functions of Lum/Ce6@PLGA nanoparticles. A) Schematic illustration of the synthesis of Lum/Ce6@PLGA nanoparticles. B) A TEM image of Lum/Ce6@PLGA nanoparticles. C) The luminescence spectra of Lum/Ce6@PLGA nanoparticles in the absence or presence of ClO^−^. “+” means that the nanoparticles incubated with ClO^−^ for evaluating the property of BRET. D,E) Luminescence images (D) and quantitative data (E) of Lum/Ce6@PLGA nanoparticles under different conditions as indicated. F) Representative luminescence images of Lum/Ce6@PLGA nanoparticles incubated with H_2_O_2_, MPO, and Cl^−^ at different time points. G,H) Representative luminescence images (G) and the luminescence intensities (H) of Lum/Ce6@PLGA nanoparticles after addition with different concentrations of Tempol. J) Representative luminescence images (I) and the luminescence intensities (J) of Lum/Ce6@PLGA nanoparticles after addition with different concentrations of 4‐ABAH. K,L) Representative flow cytometry graphs (K) and statistical data (L) of the percentages of apoptotic neutrophils after treatment with Lum/Ce6@PLGA nanoparticles under different conditions as indicated for 30 min. As the isolated neutrophils were kept in D‐hanks solution containing NaCl, no additional Cl^−^ was added into each group for this experiment. M,N) Representative images (M) and the statistic result (N) of *S. aureus* colonization at 30 min after incubation with different conditions as indicated. Data are presented as mean ± SEM. Statistical significance was calculated by one‐way ANOVA with Tukey's post hoc test.

Considering the high levels of neutrophils, MPO, and H_2_O_2_ in the inflammatory site, Lum/Ce6@PLGA nanoparticles with BRET property were designed to target and regulate the inflammatory microenvironment (**Figure** **3**A). In this system, luminol (Lum) and chlorin e6 (Ce6) at different molar ratios were first co‐encapsulated into poly (lactic‐co‐glycolic acid) (PLGA) nanoparticles by oil‐in‐water (O/W) emulsion. The molar ratio of Ce6: luminol at 1:0.5, was chosen to synthesize the following Lum/Ce6@PLGA nanoparticles, considering the highest encapsulation efficiency of Ce6 at the tested conditions (Figure S1A, Supporting Information). As shown in transmission electron microscope (TEM) images, the obtained Ce6@PLGA, Lum@PLGA, and Lum/Ce6@PLGA nanoparticles had uniform spherical structures (Figure 3B; Figure S1B,C, Supporting Information). The average diameter of Lum/Ce6@PLGA nanoparticles was ≈120 nm (Figure S1D, Supporting Information). It was demonstrated in Figure S1E (Supporting Information) that the Lum/Ce6@PLGA nanoparticles were stable in deionized water, phosphate buffer saline (PBS), and Dulbecco's modified eagle's medium (DMEM). Meanwhile, UV‐visible absorption spectra and fluorescence spectra showed that Ce6 was successfully loaded into Lum/Ce6@PLGA nanoparticles (Figure S1F,G, Supporting Information).

Next, the luminescence spectra of Lum/Ce6@PLGA nanoparticles were studied in the presence of pypocholoride (ClO^−^), which would appear in the inflammatory microenvironment. The two fluorescence emission peaks, one at 450 nm and the other at 672 nm, were found in the luminescence spectrum of Lum/Ce6@PLGA nanoparticles, while only one peak at ≈450 nm was found in the spectrum of the Ce6@PLGA + Lum@PLGA mixture (Figure 3C). Our results demonstrated that the chemical luminescence emitted from luminol in the presence of ClO^−^ could excite Ce6 in Lum/Ce6@PLGA nanoparticles by BRET to yield Ce6 fluorescence with emission at 672 nm, while such BRET phenomenon would not occur for the simple mixture of Ce6@PLGA + Lum@PLGA, suggesting that the close distance between luminol and Ce6 is critical for the BRET effect.

Next, bioluminescence imaging was carried out for Lum/Ce6@PLGA nanoparticles at different Ce6 concentrations in the presence of H_2_O_2_, MPO, and chloride ion (Cl^−^) (Figure 3D). Lum/Ce6@PLGA nanoparticles showed no detectable luminescence signal with addition of H_2_O_2_, as well as rather weak signals upon addition of both H_2_O_2_ and MPO (the purchased MPO contains a very small amount of sodium chloride). In contrast, strong luminescence signals were observed from Lum/Ce6@PLGA nanoparticles in the presence of either ClO^−^, or the mixture of H_2_O_2_, MPO, and Cl^−^ (Figure 3D,E). Our results demonstrated that ClO^−^ either from external addition or from the MPO‐triggered oxidization of Cl^−^ by H_2_O_2_, is primarily responsible for the generation of luminescence signals from luminol. By monitoring the luminescence signals of different samples over time, we found that compared to the direct addition of external ClO^−^, the mixture of H_2_O_2_, MPO and Cl^−^ triggered more sustained generation of luminescence from Lum/Ce6@PLGA nanoparticles (Figure 3F; Figure S2C–E, Supporting Information), likely owing to the fact that ClO^−^ would be continuously generated from MPO‐triggered oxidization of Cl^−^ in this sample. Moreover, the bioluminescence signals from Lum/Ce6@PLGA nanoparticles were gradually enhanced with the increasing concentration of Ce6 (Figure S2A,B, Supporting Information).

To further prove the specificity of bioluminescence generated from Lum/Ce6@PLGA nanoparticles, Tempol, a ROS scavenger, and 4‐ABAH, a kind of MPO inhibitor, were used to block H_2_O_2_ and MPO, respectively. The results of bioluminescence imaging and the correlation analysis showed that the luminescence intensities of Lum/Ce6@PLGA nanoparticles and the concentrations of inhibitors were in negative correlations (Tempol: r = −7826, P < 0.0001; 4‐ABAH: r = −9392, P < 0.0001), demonstrating the good specificity of this reaction (Figure 3G–J; Figure S2F,G, Supporting Information).

The generation of singlet oxygen (^1^O_2_) induced by BRET in the Lum/Ce6@PLGA nanoparticles was further evaluated. It was found in Figure S3 (Supporting Information) that ^1^O_2_ could be continuously produced by Lum/Ce6@PLGA nanoparticles in the presence of H_2_O_2_, MPO, and Cl^−^, but not in the presence of only H_2_O_2_, consistent to our bioluminescence imaging results. Our results indicated that luminol could be used as an effective luminescence donor to produce bioluminescence in inflammation sites with abundant H_2_O_2_, MPO, and Cl^−^, subsequently exciting the photosensitizer Ce6 to produce cytotoxic ^1^O_2_ via BRET.

Neutrophils in responsive to inflammatory signals would be quickly recruited to the inflammatory lesion. While contributing to effectively killing of pathogens, excessive neutrophils in the lesion would further aggravate the inflammatory condition, leading to additional organ damage.^[^ ^33^, ^34^ ^]^ Recently, it has been reported that the depletion of inflammatory neutrophils could promote the recovery of acute lung injury.^[^ ^35^, ^36^, ^37^ ^]^ However, considering the importance of neutrophils in the innate immunity, an innovative approach to selectively kill excessive neutrophils only in the inflammatory lesion but not those in normal organs would be of great interests in combating inflammatory and infectious diseases. We hypothesize that our Lum/Ce6@PLGA nanoparticles, being non‐toxic under normal physiological environment, can generate cytotoxic ^1^O_2_ via BRET in the presence of H_2_O_2_ and MPO elevated in the inflammatory lesion, and thus would be an ideal therapeutic agent to selective deplete excessive neutrophils in the inflammatory conditions. Under physiologic conditions, macrophages survive longer than neutrophils. Furthermore, compared to macrophages, it is known that neutrophils are more susceptible to oxidative stress.^[^ ^38^, ^39^ ^]^Therefore, as shown in our in vitro experiments, Lum/Ce6@PLGA nanoparticles, showing no appreciable cytotoxicity to neutrophils and different types of cells under normal culture conditions, could dramatically promote neutrophils rather than macrophages apoptosis in the presence of H_2_O_2_, MPO, and Cl^−^ (Figure 3K,L; Figures S4 and S5, Supporting Information). Furthermore, in the presence of H_2_O_2_, MPO, and Cl^−^, it was found that Lum/Ce6@PLGA nanoparticles could decrease the levels of pro‐inflammatory cytokines including IL‐6 and TNF‐α by inducing the apoptosis of neutrophils (Figure S6, Supporting Information). Meanwhile, NETosis is known as specific form of neutrophil death where neutrophil extracellular traps (NETs) are formed to aggravate the level of inflammation. Therefore, the NETosis assay was performed to evaluate the level of NETs by quantifying the amount of double stranded DNA (dsDNA). It was found in NETosis assay that our Lum/Ce6@PLGA nanoparticles could not significantly cause NETosis of the neutrophils in the inflammatory microenvironment (Figure S7, Supporting Information). Such results suggest that Lum/Ce6@PLGA nanoparticles may be able to remove excessive neutrophils in the inflammatory microenvironment to potentially block inflammation and reduce inflammatory injury.

The bactericidal effect of Lum/Ce6@PLGA nanoparticles was then assessed in vitro. *S. aureus* (3 × 10^4^ CFU) was incubated with Lum/Ce6@PLGA nanoparticles in presence of H_2_O_2_, MPO, and Cl^−^ for 30 min and cultured on agar plates. In the presence of H_2_O_2_ + MPO + Cl^−^, which by itself showed no antibacterial effect, Lum/Ce6@PLGA nanoparticles could significantly reduce the number of *S. aureus*, presenting more significant antibacterial effect compared to the mixture of Lum@PLGA + Ce6@PLGA under the same condition (Figure 3M,N; Figure S8A,B, Supporting Information). However, the bactericidal effect of Lum/Ce6@PLGA nanoparticles was diminished when Tempol or 4‐ABAH was added (Figure S9A,B, Supporting Information). Furthermore, confocal fluorescence images confirmed that Lum/Ce6@PLGA nanoparticles in the presence of H_2_O_2_ + MPO + Cl^−^ could kill the majority of treated bacteria (Figure S10, Supporting Information). Our results suggest that Lum/Ce6@PLGA nanoparticles could be a potential antibacterial agent in responsive to H_2_O_2_ and MPO, owing to the BRET‐based generation of ^1^O_2_ to kill bacteria.

### 2.3 In Vivo Protective Effects against LPS‐Induced Peritonitis by Lum/Ce6@PLGA

**Figure 4:**
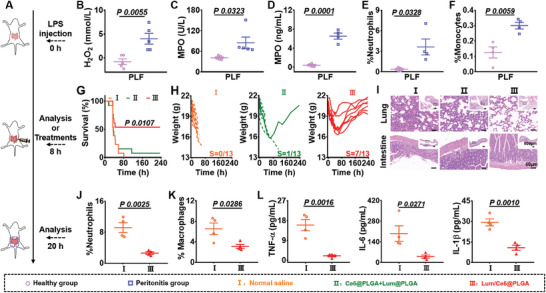
In vivo protective effects against LPS‐induced peritonitis by Lum/Ce6@PLGA nanoparticles. A) A schematic illustration of LPS‐induced peritonitis model and treatment with Lum/Ce6@PLGA nanoparticles. B) The concentrations of H_2_O_2_ in PLF of peritonitis mice at 8 h after challenge (n = 5). C,D) The activities (C) and concentrations (D) of MPO in PLF at 8 h after challenge (n = 4 or 5). E,F) The statistical data of the proportions of neutrophils (E) and monocytes (F) in PLF at 8 hours after LPS challenge (n = 5). G,H) Survival rate (G) and individual body weights (H) of the LPS‐treated mice after different treatments (n = 13). I) The H&E staining images of lung and intestine sections collected from mice at 12 h after different treatments. J,K) The proportions of neutrophils (J) and macrophages (K) in PLF after treatments (n = 4). L) Cytokine levels including TNF‐α, IL‐6, and IL‐1β in PLF collected at 12 h after different treatments (n = 4). Data are presented as mean ± SEM. The statistical significance between two groups was calculated by two‐sided Student's t‐test.

In sepsis and acute lung injury, neutrophils are overactivated to release a large number of toxic mediators to aggravate tissue damages.^[^ ^40^ ^]^ Therefore, based on the in vitro results, we hypothesized that the ^1^O_2_ generated by Lum/Ce6@PLGA nanoparticles in the inflammatory microenvironment may promote neutrophil apoptosis, reduce inflammation, and promote the recovery of diseases. To verify our hypothesis, lipopolysaccharide (LPS)‐induced peritonitis, a model of aseptic inflammation induced by intraperitoneal (i.p.) injection of LPS, was constructed to evaluate the treatment effect of Lum/Ce6@PLGA nanoparticles (**Figure** **4**A). As shown in Figures 4B–F and S11A,B (Supporting Information), the concentrations of H_2_O_2_ and MPO, as well as the proportion of immune cells such as neutrophils and monocytes, were significantly increased at 8 h after i.p. injection of LPS, indicating the establishment of aseptic inflammation.

For the treatment experiment, Lum/Ce6@PLGA nanoparticles were used by i.p. injection. Notably, Lum/Ce6@PLGA treatment could significantly promote the survival of those mice with LPS‐induced peritonitis (Figure 4G). The body weights for seven out of thirteen mice returned to normal within 10 days (Figure 4H). Meanwhile, hematoxylin‐eosin (H&E) stained organ slices demonstrated that Lum/Ce6@PLGA nanoparticles could reduce tissue damages caused by LPS‐induced peritonitis (Figure 4I; Figure S12, Supporting Information). Furthermore, the peritoneal lavage fluids (PLF) and major organs were collected to analyze the characteristics of the immune state at 12 h after the treatments. It was found that the count of total cells in PLF, as well as the percentages of neutrophils and macrophages among those cells, were significantly decreased in the Lum/Ce6@PLGA treated group (Figure 4J–K; Figure S14, Supporting Information), likely owing to generation of cytotoxic ^1^O_2_ by those BRET nanoparticles in responsive to the inflammatory microenvironment with upregulated MPO and H_2_O_2_. Interestingly, the levels of cytokines, including TNF‐α, IL‐6, and IL‐1β, were also significantly decreased in Lum/Ce6@PLGA‐treated mice (Figure 4L). Those results proved that Lum/Ce6@PLGA nanoparticles by killing excessive inflammation‐related immune cells such as neutrophils in the inflammatory lesion would lead to down‐regulated levels of cytokines and reduced organ damages, further resulting in improved animal survival upon aseptic inflammation.

### 2.4 In Vivo Therapeutic Effect against *S. Aureus*‐Induced Peritonitis by Lum/Ce6@PLGA

**Figure 5:**
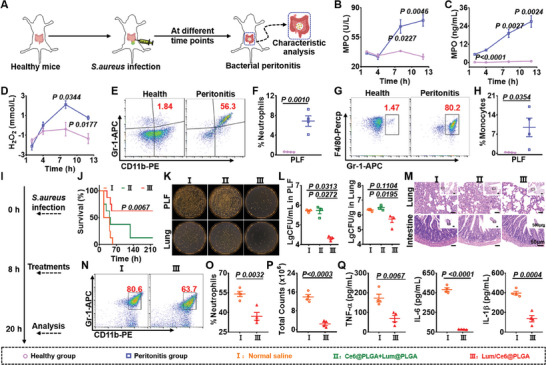
In vivo therapeutic effect against *S. aureus*‐induced peritonitis by Lum/Ce6@PLGA nanoparticles. A) A schematic illustration of *S. aureus*‐induced peritonitis model. B<C) The activities (B) and concentrations (C) of MPO in PLF at different time points in the peritonitis mice (n = 3). D) The concentrations of H_2_O_2_ in PLF at different time points (n = 3). E–H) Representative flow cytometry graphs and statistical data of the proportions of neutrophils (E&F) and monocytes (G,H) in PLF collected from the healthy and peritonitis mice at 8 hours after *S. aureus* infection (n = 4). I) A schematic illustration of experimental schedule for the treatments of *S. aureus*‐induced peritonitis. J) Survival rate of the peritonitis mice after different treatments (n = 8). K,L) Representative images (K) and the statistic data (L) of *S. aureus* colonization in PLF and lung of the mice at 12 h after different treatments as indicated (n = 3). M) The H&E staining images of lung and intestine sections collected from mice at 12 h after different treatments. N,O) Representative flow cytometry graphs (N) and statistical data (O) of the proportion of neutrophils in the PLF at 12 h after different treatments (n = 4). P) The total number of cells in PLF after different treatments. Q) Cytokine levels including TNF‐α, IL‐6, and IL‐1β in the PLF collected from the peritonitis mice at 12 h after different treatments as indicated (n = 4). Data are presented as mean ± SEM. The statistical significance between two groups was calculated by two‐sided Student's t‐test. More than two groups were calculated by one‐way ANOVA with Tukey's post hoc test.

Next, the in vivo anti‐infection therapeutic effect of Lum/Ce6@PLGA nanoparticles was studied in various infectious models. *S. aureus*‐induced peritonitis is a serious complication in peritoneal dialysis patients.^[^ ^41^ ^]^ The *S. aureus*‐induced peritonitis was established by i.p. injection of *S. aureus* (**Figure** **5**A). It was found that the levels of MPO in the PLF were significantly up‐regulated with the progress of peritonitis (Figure 5B,C). Furthermore, we detected H_2_O_2_ in the lesion by photoacoustic imaging using our previously developed H_2_O_2_‐specific nanoprobe (Figure S15A, Supporting Information).^[^ ^42^ ^]^ It was shown that the concentration of H_2_O_2_ was gradually increased in abdominal cavity and reached a peak at 8 h post injection of *S. aureus* (Figure 5D; Figure S15B,C, Supporting Information). Meanwhile, the abdominal immune cells in healthy mice and mice with *S. aureus*‐induced peritonitis were analyzed at 8 h post‐infection. As expected, the percentages of monocytes and neutrophils in the PLF collected from mice with peritonitis were significantly higher than that of healthy mice (Figure 5E–H). Furthermore, it was found that Lum/Ce6@PLGA nanoparticles could promote the apoptosis of neutrophils in vivo (Figure S16, Supporting Information).

Considering the peak time of H_2_O_2_ post‐infection, the infected mice were randomly allocated into normal saline group, Ce6@PLGA + Lum@PLGA group or Lum/Ce6@PLGA group and then intraperitoneally injected with the corresponding nanoparticles at 8 h after *S. aureus* infection (Figure 5I). Remarkably, 62.5% of mice with *S. aureus*‐induced peritonitis survived after treatment with Lum/Ce6@PLGA nanoparticles, in marked contrast the 10% mouse survival in normal saline‐treated group and 12.5% mouse survival in Ce6@PLGA+Lum@PLGA‐treated group (Figure 5J). Furthermore, the survived mice post Lum/Ce6@PLGA treatment could return to normal body weights within 9 days (Figure S17, Supporting Information).

The bacteria load in the major organs of the treated mice was then studied at 12 h after various treatments (Figure 5I). Notably, the bacteria count in PLF, lung, and liver of Lum/Ce6@PLGA‐treated mice showed obvious decrease in comparison with other groups (Figure 5K–L; Figure S18A,B, Supporting Information), evidencing the in vivo bacterial clearance capability of Lum/Ce6@PLGA in the infectious lesion. H&E staining of major organ slices further proved that Lum/Ce6@PLGA could remarkably alleviate intestinal and lung injury induced by *S. aureus* infection (Figure 5M; Figure S19, Supporting Information). Simultaneously, the percentages of neutrophils and the counts of the total cells in PLF were also decreased in the Lum/Ce6@PLGA‐treated group (Figure 5N–P; Figure S20A–D, Supporting Information). Furthermore, the levels of various proinflammatory cytokines, including TNF‐α, IL‐6, and IL‐1β, were significantly decreased in the mice treated by Lum/Ce6@PLGA (Figure 5Q). These results proved that the Lum/Ce6@PLGA nanoparticles could efficiently protect the mice against *S. aureus*‐induced peritonitis.

### 2.5 In Vivo Therapeutic Effects against CLP‐Induced Peritonitis by Lum/Ce6@PLGA

**Figure 6:**
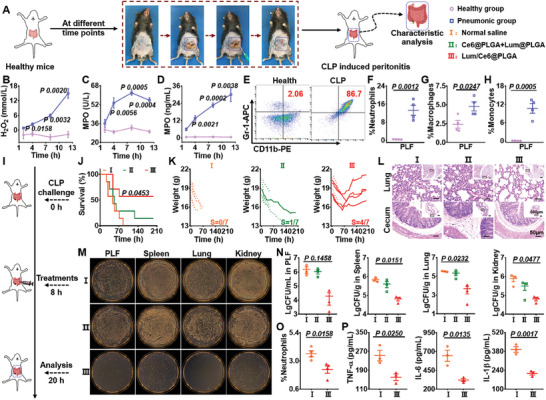
In vivo protective effects against CLP‐induced peritonitis by Lum/Ce6@PLGA nanoparticles. A) A schematic illustration of CLP‐induced peritonitis model. B) The concentration of H_2_O_2_ in PLF at different time points (n = 3). C,D) The activities (C) and concentrations (D) of MPO in PLF at different time points in the mice with CLP operation (n = 3). E–H) Representative flow cytometry graphs and statistical data of the proportions of neutrophils (E&F), macrophages (G) and monocytes (H) in PLF collected from healthy and peritonitis mice at 8 hours after CLP operation (n = 4). I) A schematic illustration of experimental schedule for the treatments of CLP‐induced peritonitis. J,K) Survival rate (J) and individual body weights (K) of the peritonitis mice after different treatments (n = 7). L) The H&E staining images of lung and cecum sections collected from mice at 12 h after different treatments. M,N) Representative images (M) and the statistic data (N) of bacteria colonization in PLF and major organs of mice at 12 h after different treatments as indicated (n = 3). O) The proportion of neutrophils in the PLF at 12 h after different treatments. P) Cytokine levels including TNF‐α, IL‐6, and IL‐1β in the PLF collected from the peritonitis mice at 12 hours after different treatments as indicated (n = 3). Data are presented as mean ± SEM. The statistical significance between two groups was calculated by two‐sided Student's t‐test. More than two groups were calculated by one‐way ANOVA with Tukey's post hoc test.

Cecal ligation and puncture (CLP), as a model of multiple infections and sepsis, can represent the complexity of human infections with polymicrobial critical infections and inflammation.^[^ ^43^ ^]^ It has been shown that the microenvironment of polymicrobial infections would recruit neutrophils to aggravate the progress of inflammation and finally exacerbates multiorgan dysfunction.^[^ ^44^ ^]^ Therefore, the changes of the microenvironment in CLP‐induced peritonitis were first studied, which was established by exposing and perforating the cecum (**Figure** **6**A). The changes of H_2_O_2_ levels in the abdominal cavity, the infectious site, were evaluated by photoacoustic imaging using our H_2_O_2_‐specific nanoprobe (Figure 6A; Figure S23A, Supporting Information). It was revealed that the concentration of H_2_O_2_ in abdominal cavity was gradually increased with the exacerbated CLP‐induced peritonitis (Figure 6B; Figure S23B,C, Supporting Information). Consistently, the levels of MPO were also significantly increased with the aggravation of CLP model (Figure 6C,D). At 8 h post CLP challenge, the PLF from healthy and CLP‐treated mice were harvested to analyze immune cells in abdominal cavity by flow cytometry. It was shown that the percentages of neutrophils, monocytes, and macrophages were significantly increased in the peritonitis mice compared to those in healthy mice (Figure 6E–H; Figure S24A,B, Supporting Information).

Next, we used Lum/Ce6@PLGA nanoparticles to treat CLP‐treated mice (Figure 6I). It was found that CLP‐treated mice by i.p. injection with Lum/Ce6@PLGA could achieve a survival rate of 57.1%, which was much higher compared to 0% survival rate for saline treated mice and 14.3% survival rate for Ce6@PLGA + Lum@PLGA treated mice (Figure 6J). The body weights of survived mice in the Lum/Ce6@PLGA group could be back to the healthy level after 9 days of infection (Figure 6K). Furthermore, as shown by microscopic images of H&E‐stained organ slices, Lum/Ce6@PLGA treatment could largely recuse the damages in lung and intestine of CLP‐treated mice (Figure 6L; Figure S25, Supporting Information).

Subsequently, the main organs and PLF of the treated mice in each group were collected, photographed and analyzed by a colony counter at 12 h after CLP challenge. The numbers of bacterial colonies in lung, kidney, spleen and PLF in the mice treated with Lum/Ce6@PLGA were significantly decreased (Figure 6M,N; Figure S26A,B, Supporting Information), indicating that Lum/Ce6@PLGA could effectively eliminate bacteria in CLP‐treated mice. To investigate the role of Lum/Ce6@PLGA nanoparticles in vivo, the changes of the immune status were evaluated in details (Figure 6I). It was displayed that the proportion of neutrophils in the Lum/Ce6@PLGA‐treated mice was significantly down‐regulated (Figure 6O), while the percentages of monocytes, macrophages and the total cells in PLF showed no significant change (Figures S27 and S28, Supporting Information). In consistent with the change of immune cells, the levels of pro‐inflammatory cytokines in the abdominal cavity, including TNF‐α, IL‐6 and IL‐1β, were markedly decreased compared with those of saline treated group (Figure 6P). These results demonstrated that treatment with Lum/Ce6@PLGA nanoparticles could effectively modulate the inflammatory status in the CLP model with polymicrobial infections, by reducing the infiltration of immune cells and decreasing the secretion of cytokines in the lesion.

### 2.6 In Vivo Therapeutic Effects against Pneumonia by Lum/Ce6@PLGA

**Figure 7:**
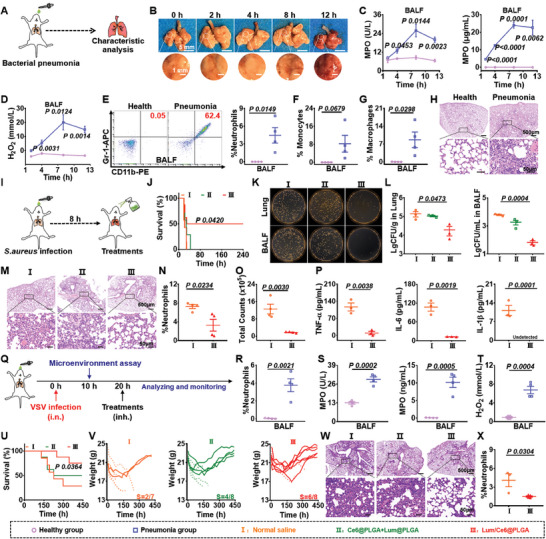
In vivo protective effects against pneumonia by Lum/Ce6@PLGA nanoparticles. A) A schematic illustration of *S. aureus*‐induced pneumonia model. B) Photographs of lung tissues in the mice at different time points. C) The activities and concentrations of MPO in BALF of pneumonia mice at different time points in the pneumonia mice (n = 3). D) The concentrations of H_2_O_2_ in BALF of pneumonia mice at different time points (n = 3). E–G) Representative flow cytometry graphs and statistical data of the proportions of neutrophils (E), monocytes (F), and macrophages (G) in BALF collected from the healthy and pneumonia mice at 8 hours after infection (n = 4). H) The H&E staining images of lung tissues collected at 8 hours after *S. aureus* infection. I) A schematic illustration of experimental schedule for the treatments of *S. aureus*‐induced pneumonia with inhalation administration of Lum/Ce6@PLGA nanoparticles. J) Survival rate of the peritonitis mice after different treatments (n = 7 or 8). K,L) Representative images (K) and the statistic data (L) of *S. aureus* colonization in BALF and lung tissue of the mice at 12 h after different treatments (n = 3). M) The H&E staining images of the lung tissues collected from mice at 12 h after different treatments. N) The statistical data of the proportions of neutrophils in BALF collected from the pneumonic mice at 12 h after different treatments (n = 4). O) The total number of cells in BALF after different treatments (n = 4). P) Cytokine levels including TNF‐α, IL‐6, and IL‐1β in BALF collected from the pneumonia mice at 12 h after different treatments as indicated (n = 3). Q) A schematic illustration of VSV‐induced pneumonia model. i.n. means intranasal administration, inh. means inhalation administration. R) The statistical data of the proportion of neutrophils in BALF (n = 4). S) The activities and concentrations of MPO in BALF at 8 h post‐infection (n = 4). T) The concentrations of H_2_O_2_ in BALF of pneumonia mice at 8 h post‐infection. U,V) Survival rate (U) and individual body weights (V) of the pneumonia mice after different treatments (n = 7 or 8). W) The H&E staining images of the lung collected from mice at 12 h after different treatments. X) The statistical data of the proportions of neutrophils at 12 h in BALF after different treatments (n = 3). Data are presented as mean ± SEM. The statistical significance between two groups was calculated by two‐sided Student's *t*‐test. More than two groups were calculated by one‐way ANOVA with Tukey's post hoc test.

In recent years, it has been found that the majority of patients with sepsis were those with Gram‐positive bacterial pneumonia. The microenvironment in the pneumonia can aggravate lung tissue damage, induce acute respiratory distress syndrome and increase the mortality rate of patients.^[^ ^45^, ^46^ ^]^ Therefore, the changes of the lung with *S. aureus*‐induced pneumonia were studied in details, which was built by pipetting *S. aureus* into nostrils of mice (**Figure** **7**A). The photographs of *S. aureus*‐infected lung tissues showed the aggravated injury and hemorrhage in the progressed pneumonia (Figure 7B). The concentrations of H_2_O_2_, MPO and total proteins in lung tissue and bronchoalveolar lavage fluid (BALF) were also significantly increased post *S. aureus* infection (Figure 7C,D; Figures S29A,B and S30, Supporting Information). Furthermore, a large number of inflammatory cells including neutrophils, monocytes and macrophages, were found in lung tissues and BALF collected from infected mouse lungs (Figure 7E–G; Figures S31A,B and S32A–C, Supporting Information). H&E staining of lung tissues showed obvious injury and inflammatory cell infiltration in the infected lung (Figure 7H), further evidencing the successful establishment of *S. aureus*‐induced pneumonia.

Next, we used inhalation administration of Lum/Ce6@PLGA nanoparticles to treat mice with *S. aureus*‐induced pneumonia (Figure 7I). The pneumonia mice randomly were divided into normal saline group, Ce6@PLGA + Lum@PLGA group and Lum/Ce6@PLGA group with corresponding doses. It was found that Lum/Ce6@PLGA nanoparticles could effectively improve the survival of *S. aureus*‐induced pneumonia mice (Figure 7J). After 10 days of infection, the body weights of the survived mice could be back to normal (Figure S33, Supporting Information). Meanwhile, it was found that compared with the saline group and Ce6@PLGA + Lum@PLGA group, Lum/Ce6@PLGA‐treated mice had the lowest number of *S. aureus* colonies in lung and BALF (Figure 7K), as well as obviously alleviated organ damages (Figure 7M; Figure S35, Supporting Information). It was further found that the percentages of neutrophils, monocytes and macrophages in BALF and lung tissues were remarkedly decreased in the Lum/Ce6@PLGA‐treated mice (Figure 7N; Figures S36A–C and S37A–F, Supporting Information). The total number of cells in BALF was also significantly down‐regulated after treatment with Lum/Ce6@PLGA (Figure 7O). Similarly, the levels of TNF‐α, IL‐6, and IL‐1β were obviously reduced in mice treated with Lum/Ce6@PLGA nanoparticles (Figure 7P), implying effective suppression of inflammation.

In addition to bacterial pneumonia, viral pneumonia is also a kind of pulmonary infectious disease with high morbidity, which often lacks effective treatment.^[^ ^47^ ^]^We thus tested the possibility of using Lum/Ce6@PLGA nanoparticles to treat mice with vesicular stomatitis virus (VSV)‐induced pneumonia by enhancing the apoptosis of neutrophils and decreasing the level of inflammation (Figure 7Q). The characteristics of the VSV‐infected microenvironment were first analyzed. It was found that the proportion of neutrophils in BALF and lung tissue was notably increased at 8 h after infection, accompanied by the increased concentrations of H_2_O_2_ and MPO. (Figure 7R–T; Figures S38–S41, Supporting Information). Furthermore, the VSV infection could induce lung tissue damages (Figure S42, Supporting Information). Interestingly, inhalation administration of Lum/Ce6@PLGA nanoparticles could reduce the levels of neutrophils and tissue damage in mice with VSV‐induced pneumonia (Figure 7W–X; Figures S43, S44A–E, S45A–C, and S46, Supporting Information). Importantly, Lum/Ce6@PLGA nanoparticles could significantly rescue and increase the survival of the infected mice (Figure 7U,V). It is well known that the nucleic acid of virus is vulnerable to excessive ROS.^[^ ^48^ ^]^ Furthermore, it has been reported that the apoptosis of the virus‐infected cells can promote antiviral protection.^[^ ^49^ ^]^ Therefore, our results indicated that Lum/Ce6@PLGA nanoparticles might not only inhibit inflammation, but also provide the protection against VSV infection in vivo.

At last, the systemic safety of Lum/Ce6@PLGA nanoparticles was evaluated by the blood biochemical analysis and whole blood cell analysis. It was shown that there was no significant difference in blood biochemical and blood cell indexes between 0, 3, 7, and 15 days after i.p. injection of Lum/Ce6@PLGA nanoparticles, indicating the negligible toxicity of Lum/Ce6@PLGA nanoparticles to the mice under the tasted dose (Figures S47 and S48, Supporting Information).

## 3 Discussions and Conclusion

In the process of bacterial or viral infection infections, the interaction between the host and the pathogens can form a unique microenvironment at the infection site, which possesses many characteristics, including up‐regulated ROS, multiple protein virulence factors, increased infiltration of inflammation‐related immune cells, as well as specific enzymes secreted by pathogens or immune cells.^[^ ^50^, ^51^, ^52^ ^]^ In patients with acute appendicitis, it was found that a large number of neutrophils would migrate to the inflammatory site, accompanying by the increase of the levels of H_2_O_2_ and MPO in the inflammatory microenvironment. In particular, neutrophils, as the first line of innate immune responses, are thought to play a contradictory role in the progress of anti‐infection and anti‐inflammation treatment. On one hand, neutrophils are usually essential for bacterial clearance by the H_2_O_2_‐MPO‐Cl^−^ antibacterial system. On the other hand, excessive amounts of neutrophils in the infectious site are known to aggravate organ damages. Therefore, in the process of anti‐infection or anti‐inflammation, in addition to eliminating the pathogen invasion, reducing the excessive activated neutrophils within the inflammatory lesion is also necessary to reduce tissue damage and prevent chronic inflammation.

Considering the contradictory role of neutrophils, poly(lactic‐co‐glycolic) acid (PLGA) nanoparticles loaded with chlorine E6 (Ce6) and luminol (Lum) (Lum/Ce6@PLGA) were synthesized. In the inflammatory lesion with upregulated MPO and H_2_O_2_, the continuously generated HClO would oxidize luminol to generate light by a chemiluminescent reaction. Owing to the BRET effect, Ce6 co‐encapsulated inside such Lum/Ce6@PLGA nanoparticles would then be excited to generate cytotoxic ^1^O_2_, which not only kills bacteria in the lesion upon bacterial infection, but also promotes the apoptosis of excessive neutrophils to reduce inflammation. In the bacteria‐induced infections, including *S. aureus*‐induced peritonitis, CLP‐induced peritonitis and *S. aureus*‐induced pneumonia, Lum/Ce6@PLGA nanoparticles could achieve infection protection by accelerating bacterial clearance in major organs and inducing neutrophil apoptosis to down‐regulate the release of pro‐inflammatory cytokines. Meanwhile, such Lum/Ce6@PLGA nanoparticles are also capable of reducing non‐bacterial inflammation by selectively inhibiting inflammation, presenting rather effective therapeutic performances in treating LPS‐induced peritonitis or VSV‐induced pneumonia.

Our BRET‐based Lum/Ce6@PLGA nanoparticles present unique advantages in anti‐infection and anti‐inflammation treatments. Compared to conventional antibacterial therapeutics such as antibiotics, our nanomedicine is unlikely to cause drug resistance, and would not affect gut microbes that are essential for maintaining the healthy status. On the other side, although inflammatory diseases in clinic can be controlled by immunosuppressive agents (e.g., corticosteroids such as dexamethasone), those drugs usually have many systemic immunosuppression side effects, and cannot be used in antibacterial treatment.^[^ ^53^, ^54^ ^]^ In contrast, our nanomedicine is able to selectively deplete inflammatory immune cells such as neutrophils at the infection site owing to its specific responses to MPO and H_2_O_2_ that are only found in the lesion, causing no side effect to normal tissues and little systemic toxicity. Therefore, our BRET‐based smart nanomedicine by specifically responding to the inflammatory microenvironment may pave a new avenue for safe and effective treatment against infectious inflammatory diseases. In addition, the relevant studies have demonstrated that the singlet oxygen generated by Ce6 can eliminate drug‐resistant Pseudomonas aeruginosa and its biofilm.^[^ ^55^ ^]^ Therefore, it is possible that our smart nanoplatform may be able to overcome bacterial drug resistance, which will be studied in our future work.

However, there are still limitations in current work. For instance, the treatment was performed in the early stage of infection which does not mimic the late‐stage conditions in clinic. Further studies may be necessary to evaluate the capability of our nano‐therapeutics in treating infectious diseases at different stages.

## 4 Experimental Section

### Materials

PLGA, polyvinyl alcohol (PVA), and lipopolysaccharide (LPS) were purchased from Sigma‐aldrich. Ce6 was obtained from Frontier Scientific. MPO (ab91116) was purchased from BioVision. The antibodies for flow cytometry assay were obtained from Biolegend. The MPO and H_2_O_2_ assay kit were purchased from Nanjing Jiancheng Bioengineering Institute. All the C57BL/6 mice (6‐8 weeks) were performed with the protocols approved by the Institutional Animal Care and Use Committee at Soochow University.

### Study Approval and Analysis of Clinical Cases

The obtained clinical effusion was approved by the Ethics Committee of Children's Hospital of Soochow University (approval ID: 2022CS130). The informed consent was obtained from the parents. The hydrocele and acute appendicitis were first classified according to clinical data. Furthermore, acute appendicitis was divided into acute pure appendicitis, acute purulent appendicitis, and acute gangrene appendicitis based on postoperative pathological results. After centrifugation, the pellets were collected to analyze the proportion of neutrophils by flow cytometry. The contents of MPO and H_2_O_2_ in supernatants were assessed by the MPO and H_2_O_2_ kits following their instructions.

### Synthesis and Characterization of Lum/Ce6@PLGA Nanoparticles

Lum/Ce6@PLGA nanoparticles were fabricated by the o/w single‐emulsion method. Briefly, Ce6 or luminol was dissolved in dimethyl sulfoxide (DMSO) at 20 mg mL^−1^. PLGA (25 mg/mL) was dissolved in dichloromethane and added to 800 µL PVA solution (50 mg mL^−1^). Then, the solution of Ce6 and luminol was added into the above solution by sonication for 30 min after adding 3 mL deionized water. Then the emulsion was stirred overnight at room temperature. The nanoparticles were obtained after centrifugation at 14 800 rpm for 30 min and washed twice. The Ce6@PLGA nanoparticles and Lum@PLGA nanoparticles were prepared by using the same method.

The morphology of Lum@PLGA, Ce6@PLGA, and Lum/Ce6@PLGA nanoparticles was observed by TEM. The size distribution and zeta potential of Lum/Ce6@PLGA were determined by dynamic light scattering (DLS). The encapsulation efficiency of Ce6 and luminol was assessed by ultraviolet‐visible spectrophotometer.

The fluorescence spectra of Lum/Ce6@PLGA nanoparticles were measured by a fluorescence spectrometer in the presence of ClO^−^ (100 mM). Meanwhile, for investigating the luminescent properties of Lum/Ce6@PLGA nanoparticles, different concentrations of Lum/Ce6@PLGA nanoparticles were mixed with H_2_O_2_ (200 µM), MPO (10 mU) and Cl^−^ (200 mM) in a black 96‐well plate. Additionally, the nanoparticles at the Ce6 concentration (50 µg mL^−1^) were mixed with H_2_O_2_ (50 µM), MPO (10 mU) and Cl^−^ (50 mM) in a black 96‐well plate. After mixing, the luminescence signals were detected at different time points by IVIS Lumina III. Furthermore, the signals of Lum/Ce6@PLGA nanoparticles incubated with different concentrations of Tempol or 4‐ABAH were obtained in the presence of H_2_O_2_ and MPO with Cl^−^.

### In Vitro Bactericidal Effect of Lum/Ce6@PLGA Nanoparticles

*S. aureus* was cultured on trypticase soy broth medium at 37 °C. The bacteria were collected at logarithmic growth phase by centrifugation (5000 rpm, 5 min) and washed twice with deionized water for follow‐up experiments.

For antibacterial activity assays, the obtained bacteria (3 × 10^4^ CFU), cultured in 96‐well plate, were mixed with Lum/Ce6@PLGA nanoparticles (10 µg mL^−1^) in the presence of H_2_O_2_ (200 µM), MPO (2.5 mU) and Cl^−^ (5 µM) and incubated for 30 min at room temperature. After incubation, the mixtures were diluted and cultured on agar plates for 18 h at 37 °C. In the blocking experiment, the collected bacteria were incubated with Lum/Ce6@PLGA nanoparticles in the presence of H_2_O_2_/MPO/Cl^−^ with tempol or 4‐ABAH.

To further demonstrate the antibacterial activity, the bacteria (1 × 10^7^ CFU) was mixed with Lum/Ce6@PLGA nanoparticles (10 µg mL^−1^) in the presence of H_2_O_2_ (500 µM), MPO (2.5 mU) and Cl^−^ (500 µM) for 30 min. After centrifugating (5000 rpm, 5 min) and washing twice by deionized water, the bacteria were resuspended with PI and SYTO 9 at room temperature for 15 min. After washing twice by deionized water, the bacteria were visualized by a confocal microscopy.

### In Vitro Neutrophils Apoptosis Assays

Neutrophils were collected from the whole blood of C57BL/6 according to a previous report.^[^ ^56^ ^]^ Subsequently, neutrophils were seeded into centrifuge tube at the density of 1 × 10^6^ cells mL^−1^, and incubated with Lum/Ce6@PLGA (10 µg mL^−1^) or Ce6@PLGA + Lum@PLGA nanoparticles in the presence of H_2_O_2_ (200 µM), MPO (2.5 mU) and Cl^−^ (50 µM) for 30 min. After centrifugating and washing by FACS buffer, the cells were stained using Annexin V‐FITC/PI Apoptosis detection Kit following the manuals and analyzed by flow cytometry.

### In Vivo Establishment of Infection Models

The mice in all the infection models were C57BL/6 male mice. The sample size is sufficient for statistical analyses in each model. In *S. aureus*‐induced peritonitis model, S. aureus (1.3 × 10^8^ CFU) was intraperitoneally injected into the mice. In LPS‐induced peritonitis model, LPS (35 mg kg^−1^) were intraperitoneally injected into the mice. The polymicrobial peritonitis model induced by cecal ligation and puncture (CLP) was established by the pervious literature.^[^ ^57^ ^]^ In brief, the abdomen of all the mice was first depilated by the depilatory cream. After anesthetization, the cecum of the mice was exposed, punctured by a needle, ligated below the ileocecal valve, and finally placed back to peritoneal cavity. For acute pneumonia model, the mice were pipetted with *S. aureus* (5.1 × 10^8^ CFU) or VSV (5 × 10^8^ PFU) into nostrils.

### In Vivo Assessment of the Microenvironment in Infection Models

To evaluate immune microenvironment at the site of infection, the mice were randomly divided into the healthy group and the infected group. At different time intervals after infection, the concentrations of H_2_O_2_ and MPO in the infected tissues and lavage fluid were measured according to the protocol of the kits. Additionally, immune cells in the site of infection were isolated at 8 h after the establishment of the models and subsequently incubated with anti‐CD16/32 before staining by anti‐CD11c‐FITC, anti‐CD11b‐PE, anti‐F4/80‐percp, and anti‐Gr 1‐APC. After incubation with antibody, the cells were rinsed twice with FACS buffer and then analyzed with flow cytometry.

To study the local immune status of the treated mice, those mice post different treatments including saline or Lum/Ce6@PLGA nanoparticles were sacrificed at 12 h after various treatments, with their lavage fluid and the infected tissues collected for further analysis. Immune cells were isolated and stained with anti‐CD11c‐FITC, anti‐CD11b‐PE, anti‐F4/80‐percp, and anti‐Gr 1‐APC for 45 min after blocking with anti‐CD16/32. Then the cells were analyzed by flow cytometry. To detect the cytokines in lavage fluid, the supernatant was obtained by centrifugation and measure by enzyme‐linked immunosorbent assay (ELISA) kits following standard protocol.

### In Vivo Lum/Ce6@PLGA Protected the Mice from Various Infection

To verify the therapeutic efficacy of Lum/Ce6@PLGA nanoparticles, the infected mice were randomly allocated into three groups and treated by saline, Ce6@PLGA + Lum@PLGA, or Lum/Ce6@PLGA at the Ce6 dosage of 80 µg or the luminol dosage of 8.98±0.63 µg, at 8 h post‐infection. The mortality and body weights of mice were monitored. Furthermore, at 12 h after various treatments, the bacterial counts in the lavage fluid or main organs of the treated mice were analyzed by LB agar plate.

### Statistical Analysis

All results were expressed as means ± standard error of mean (SEM). The significance of differences between two groups was calculated by Student's t test. ANOVA was used when more than two groups were calculated. Statistical differences in survival were measured by the log‐rank test.

## Conflict of Interest

The authors declare no conflict of interest.

## Supporting information

Supporting Information

## Data Availability

The data that support the findings of this study are available from the corresponding author upon reasonable request.
