# Supplementary material for: Simultaneously Controlling Inflammation and Infection by Smart Nanomedicine Responding to the Inflammatory Microenvironment
Source: Adv Sci (Weinh). 2024 Sep 3;11(39):2403934. doi: 10.1002/advs.202403934 (PMC11497003; doi:10.1002/advs.202403934)
Supplement: Supplementary file 1 — Supporting Information [file ADVS-11-2403934-s001.docx]

**Supporting Information**

**Simultaneously Controlling Inflammation and Infection by Smart Nanomedicine Responding to the Inflammatory Microenvironment**

Xinjing Lv^1+^, Jie Min^1+^, Jie Huang^1+^, Hairong Wang^1^, Song Wei^1^, Chenxiao Huang^2^, Jianfeng Dai^2^, Zhengrong Chen^1^, Huiting Zhou^1^, Yunyun Xu^1^, He Zhao^1^*, Zhuang Liu^3^*, Jian Wang^1^*

1 Children’s Hospital of Soochow University, Pediatric Research Institute of Soochow University, Suzhou, Jiangsu 215123, China

2 Institutes of Biology and Medical Sciences, Jiangsu Key Laboratory of Infection and Immunity, Soochow University, Suzhou, Jiangsu, 215123, China

3 Institute of Functional Nano & Soft Materials (FUNSOM), Jiangsu Key Laboratory for Carbon-Based Functional Materials & Devices, Soochow University, Suzhou, Jiangsu, 215123, China

* E-mail addresses: zh2021@suda.edu.cn, zliu@suda.edu.cn, wj196312@vip.163.com

+ These authors contributed equally to this work.


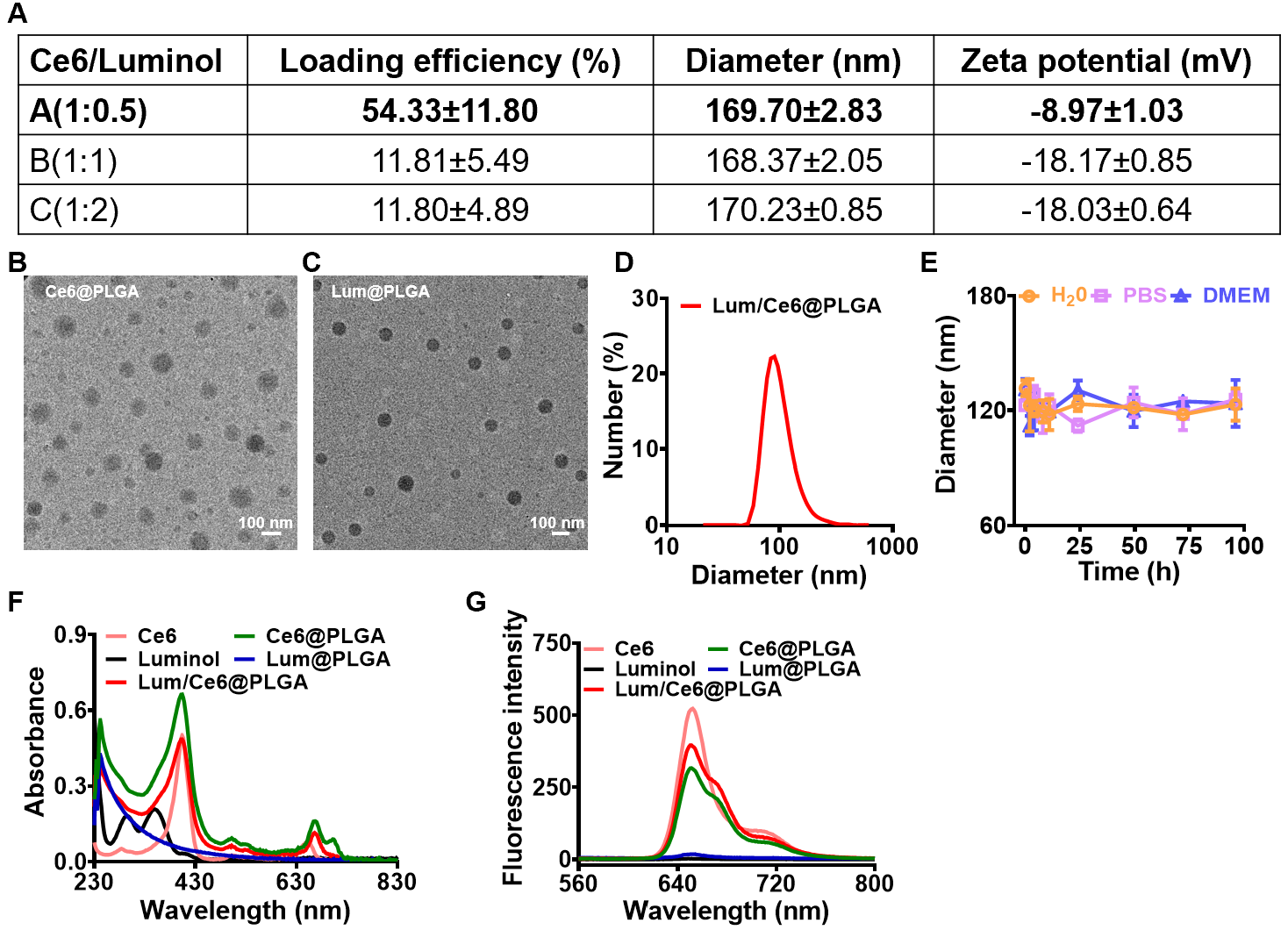


Figure S1. (A) The characterization of Lum/Ce6@PLGA nanoparticles prepared at different molar ratio of Ce6 : luminol. (B&C) TEM image of Ce6@PLGA (B) and Lum@PLGA (C) nanoparticles. (D) The DLS-measured size distribution of Lum/Ce6@PLGA nanoparticles. (E) The diameter profiles of the Lum/Ce6@PLGA nanoparticles in water, PBS or DMEM medium at different time points. (F&G) UV-visible (F) and fluorescence (G) spectra of Lum/Ce6@PLGA nanoparticles.


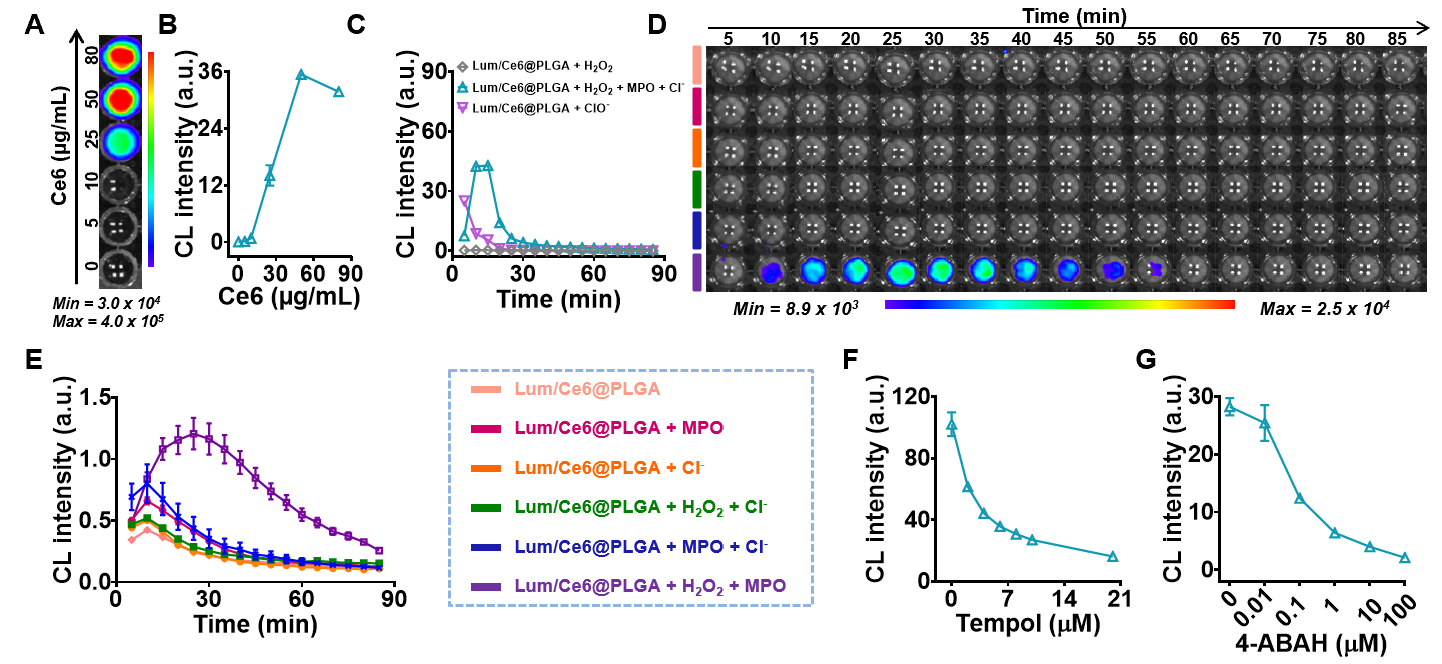


Figure S2. (A&B) Representative luminescence image (A) and quantitative data (B) of Lum/Ce6@PLGA nanoparticles with different concentrations of Ce6. (C) The quantitative data of Lum/Ce6@PLGA nanoparticles at different time points. (D&E) Representative luminescence image (D) and quantitative data (E) of Lum/Ce6@PLGA nanoparticle at different time points. (F&G) The quantitative data of Lum/Ce6@PLGA nanoparticles after addition with Tempol (F) or 4-ABAH (G). Data are presented as mean ± SEM.


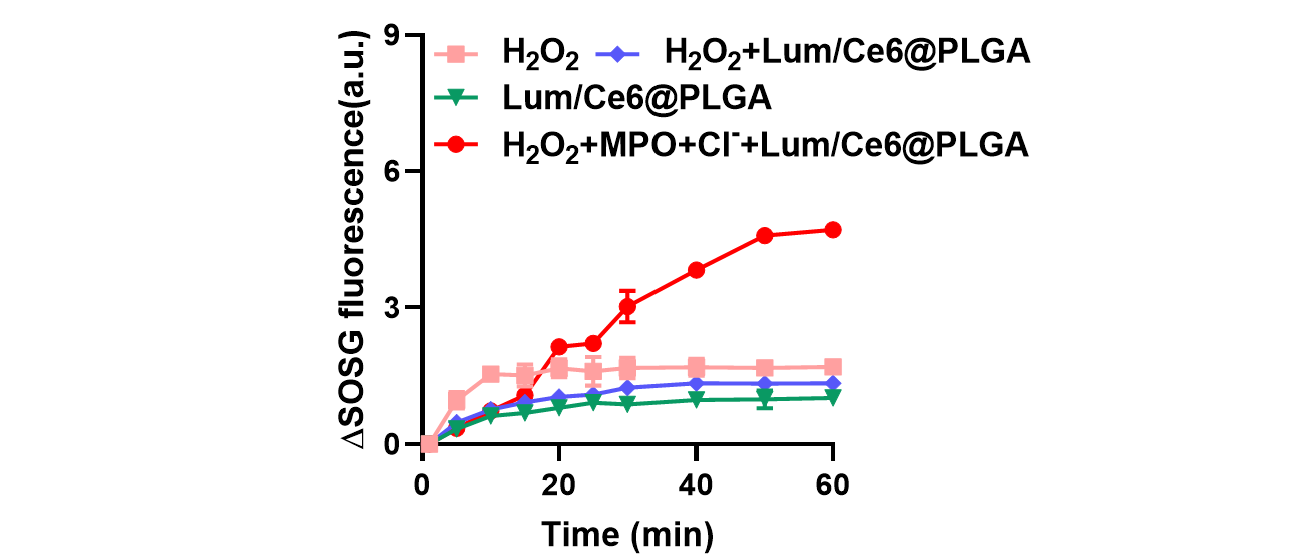


Figure S3. The generation of ^1^O_2_ by the Lum/Ce6@PLGA nanoparticles at different conditions.


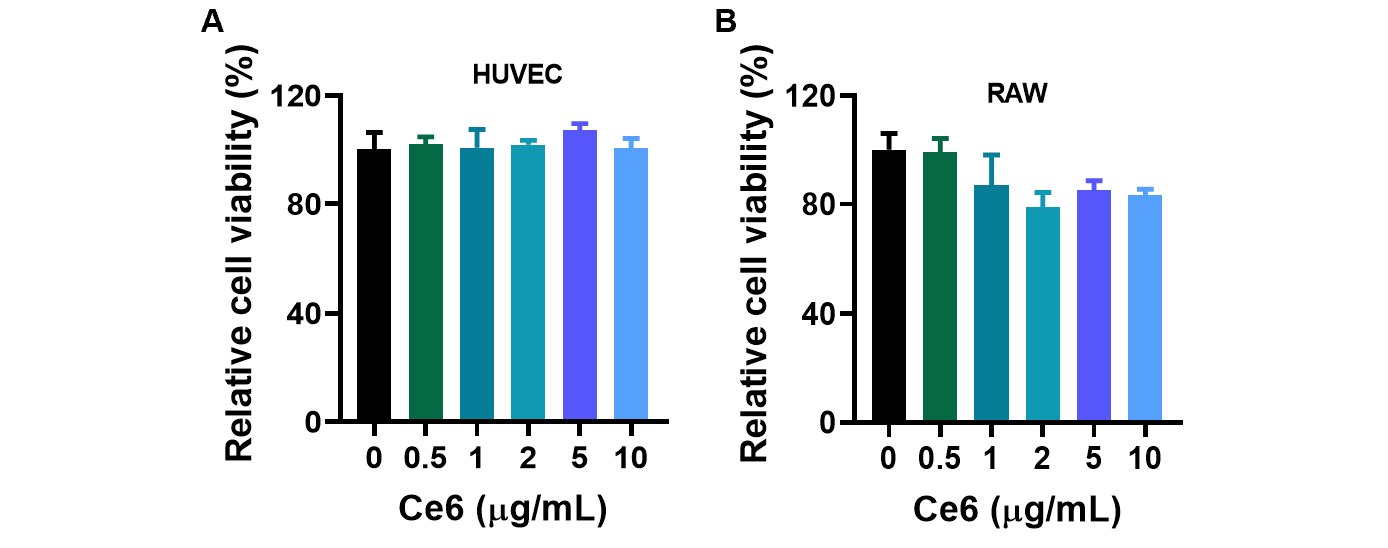


Figure S4. (A&B) The cell viabilities of HUVEC cells (A) and RAW cells (B) incubated with Lum/Ce6@PLGA nanoparticles at different concentrations of Ce6.


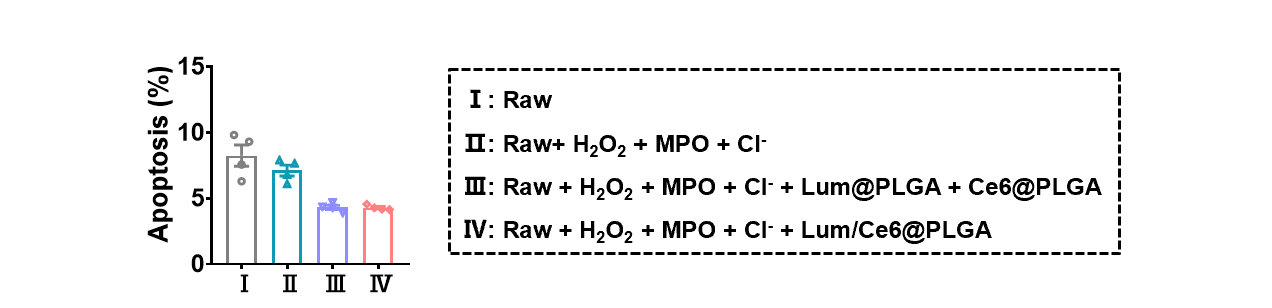


Figure S5. The statistical data of the percentages of apoptotic macrophages after treatment with Lum/Ce6@PLGA nanoparticles under different conditions as indicated for 30 min.


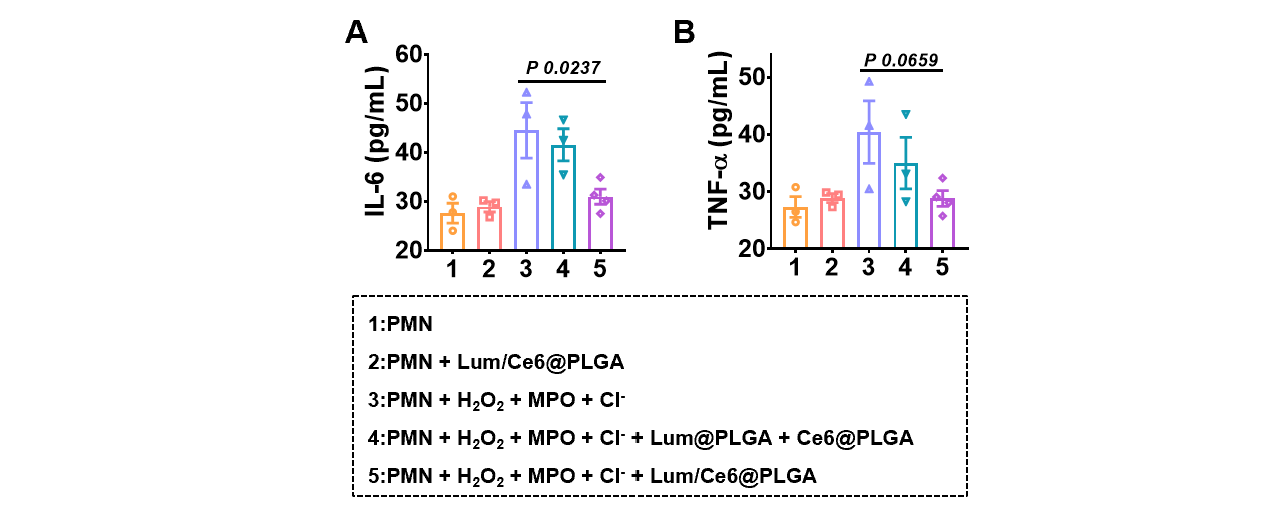


Figure S6. Cytokine levels including IL-6 (A) and TNF-α (B) after treatment with Lum/Ce6@PLGA nanoparticles under different conditions as indicated for 30 min in *vitro*.


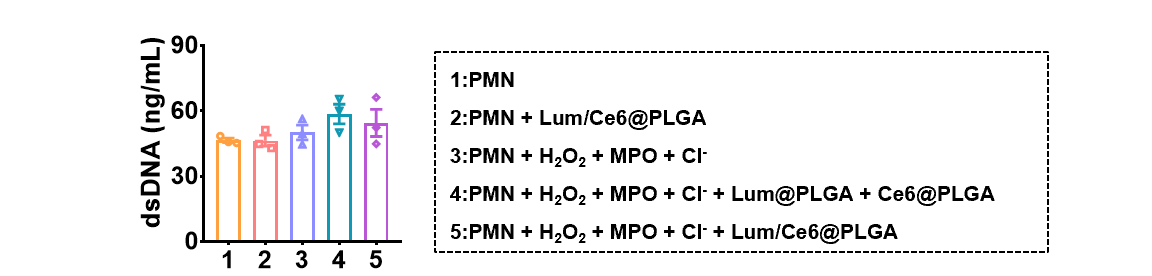


Figure S7. The concentrations of double stranded DNA (dsDNA) after treatments with Lum/Ce6@PLGA nanoparticles under different conditions.


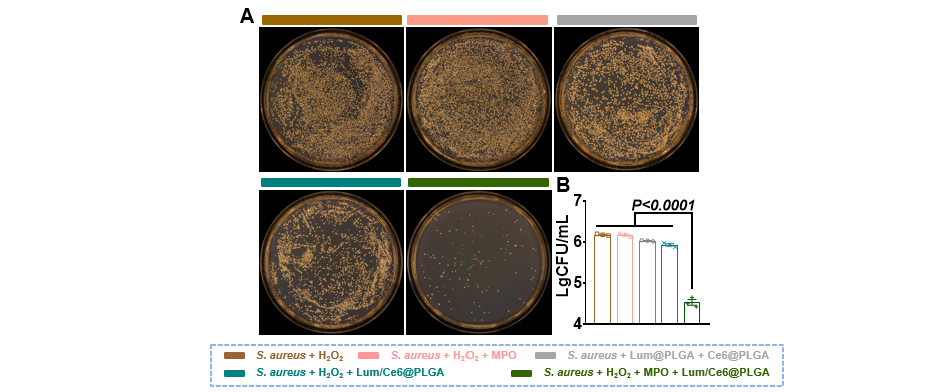


Figure S8. (A&B) Representative images (A) and the statistic result (B) of *S. aureus* colonization at 30 min after incubation with different conditions as indicated. Data are presented as mean ± SEM. Statistical significance was calculated by one-way ANOVA with Tukey’s post hoc test.


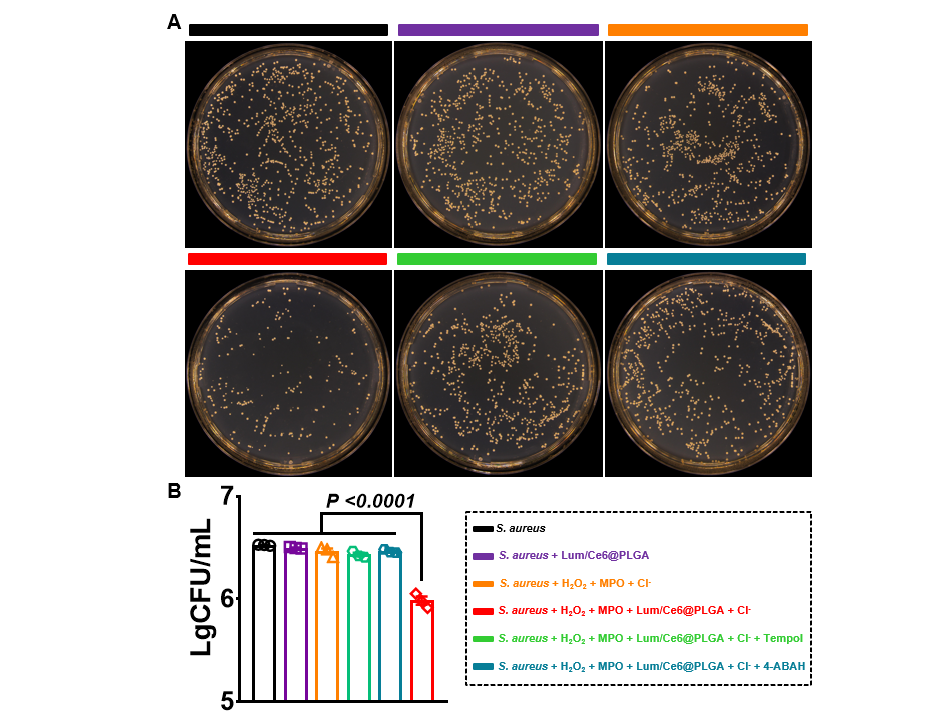


Figure S9. (A&B) Representative images (A) and the statistic results (B) of *S. aureus* colonization at 30 min after incubation with Tempol or 4-ABAH. Data are presented as mean ± SEM. Statistical significance was calculated by one-way ANOVA with Tukey’s post hoc test.


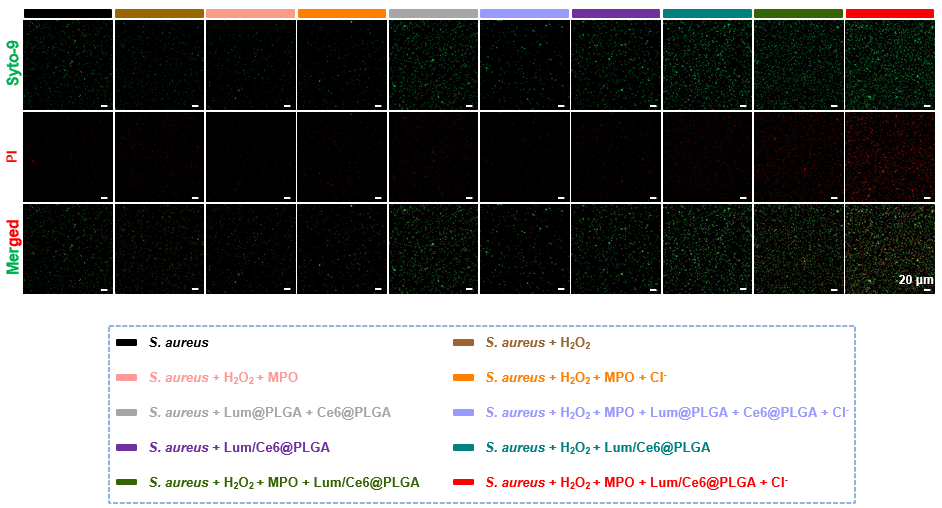


Figure S10. Confocal fluorescence microscopy images of *S. aureus* treated with Lum/Ce6@PLGA nanoparticles under different conditions as indicated for 30 min before being stained with Syto-9 (green) and PI (red), respectively.


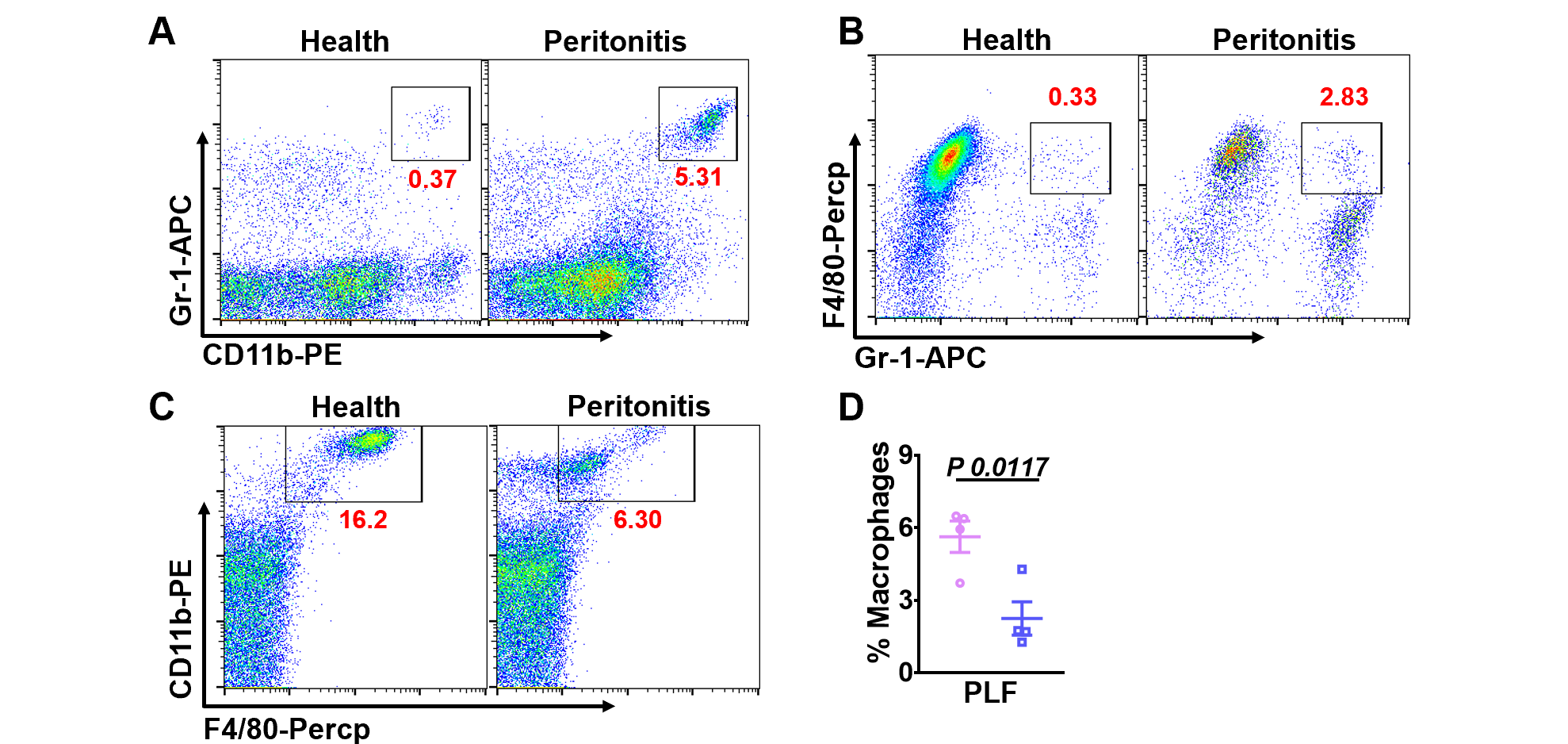


Figure S11. Representative flow cytometry graphs and statistical data of the proportions of neutrophils (A), monocytes (B) and macrophages (C&D) in PLF collected from healthy and peritonitis mice at 8 hours after LPS challenge. Data are presented as mean ± SEM. Statistical significance was calculated by two-sided Student’s t-test.


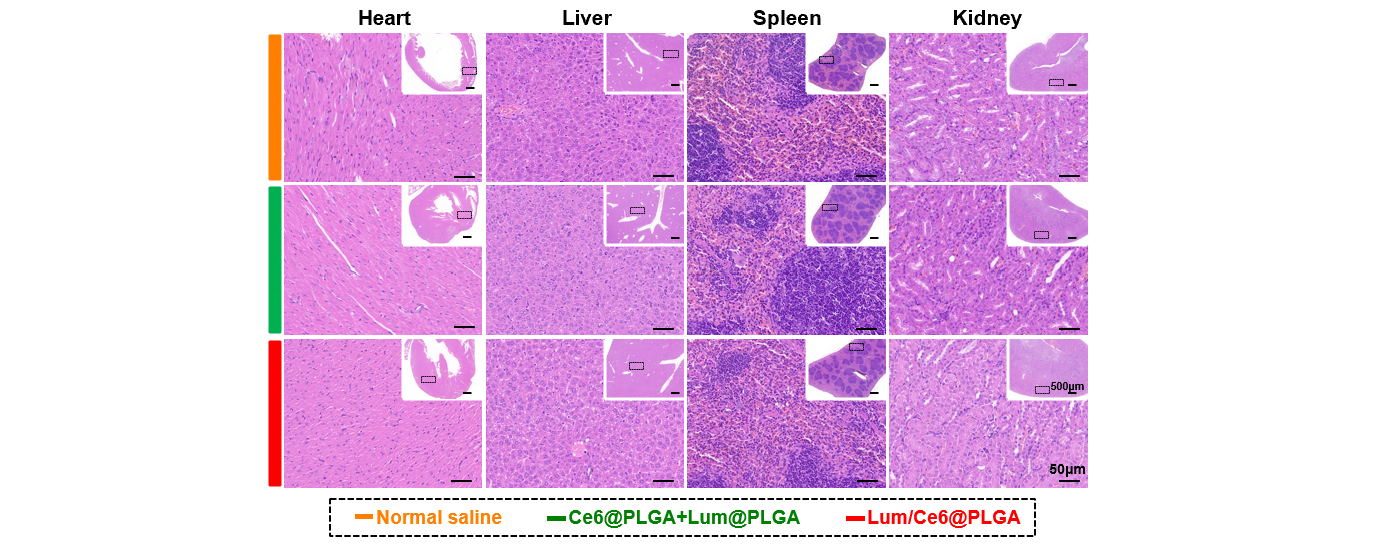


Figure S12. The H&E staining images of major organs collected from mice at 12 hours after different treatments.


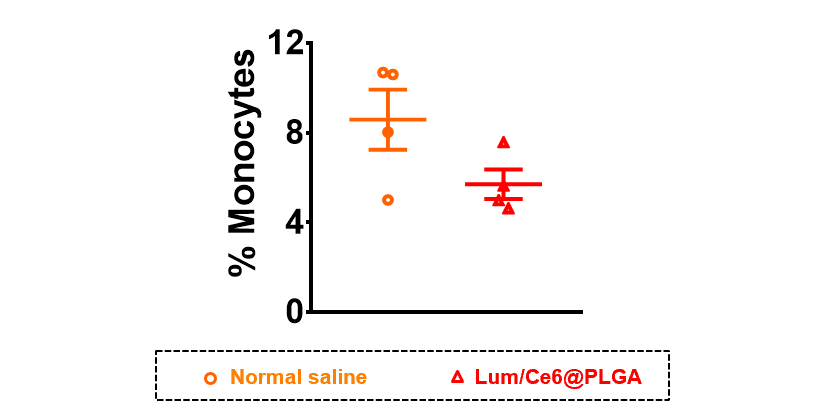


Figure S13. The statistical data of the proportions of monocytes in PLF collected from the LPS-induced peritonitis mice after different treatments. Data are presented as mean ± SEM.


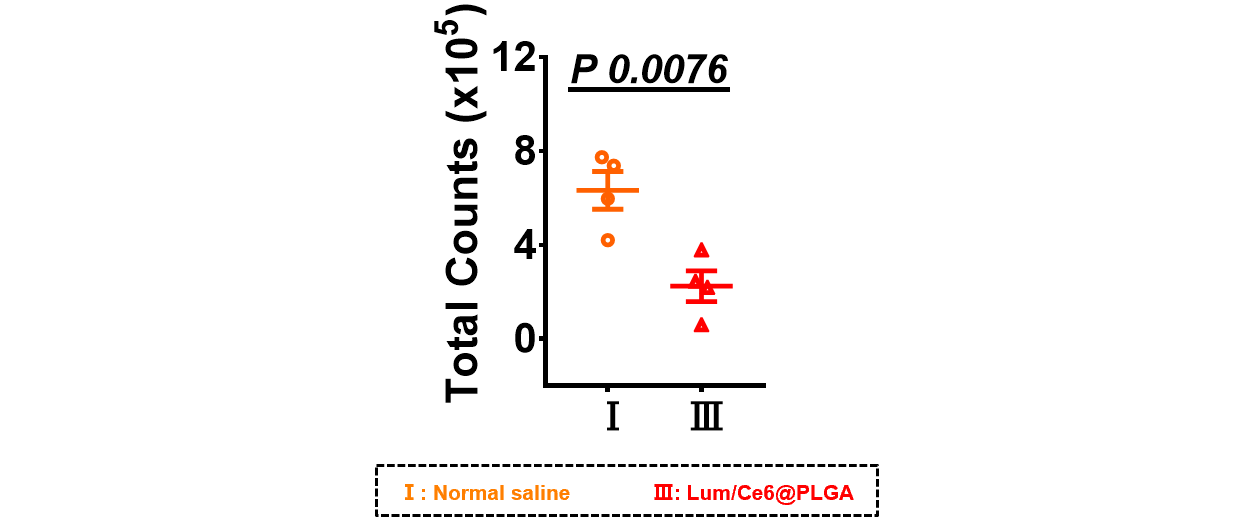


Figure S14. The total number of cells in PLF after different treatments.


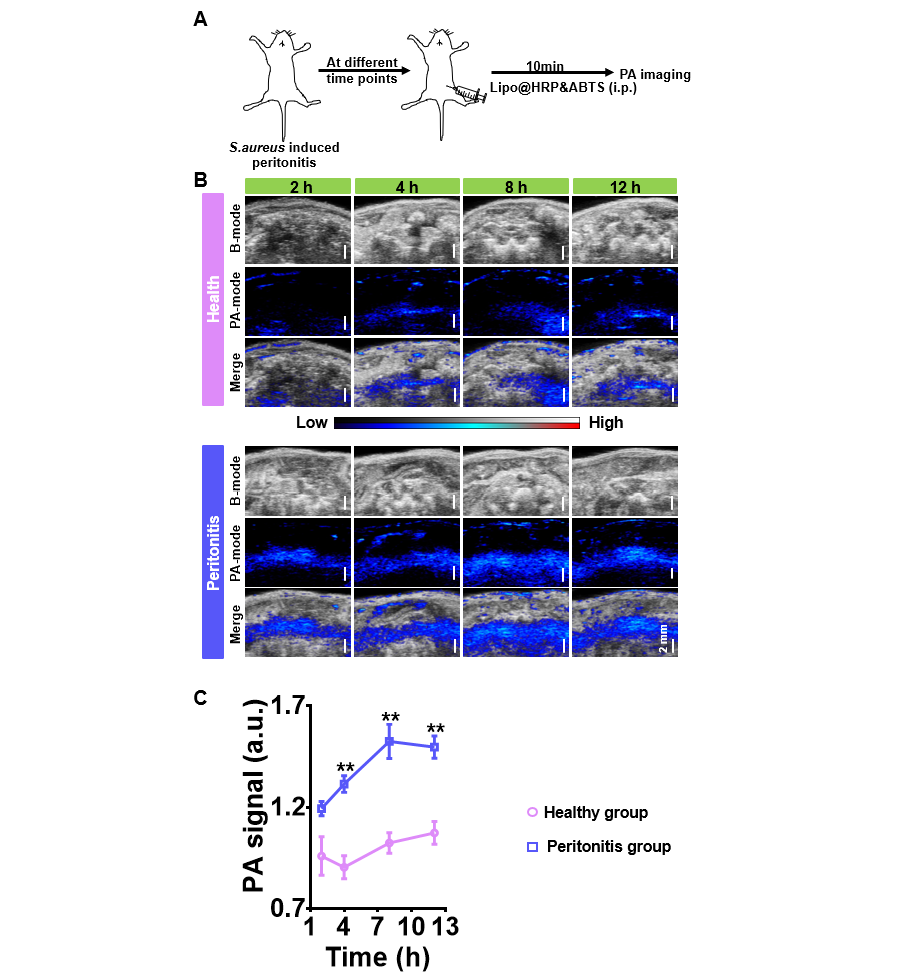


Figure S15. (A) A schematic illustration showing photoacoustic imaging of *S. aureus*-induced peritonitis for *in vivo* H_2_O_2_ detection with photoacoustic nanoprobe, Lipo@HRP&ABTS. Lipo@HRP&ABTS is a H_2_O_2_-responsive liposomal nanoprobe, which was fabricated by encapsulating horseradish peroxidase (HRP) and the substrate 2,2′-azino-bis (3-ethylbenzothiazoline-6-sulfonic acid) (ABTS).^1^ (B) *In vivo* photoacoustic images of mouse abdomen at different time points after *S. aureus*-induced peritonitis. (C) H_2_O_2_-specific photoacoustic signals for mouse abdomen of all groups based on PA imaging data in (B). Data are presented as mean ± SEM. Statistical significance was calculated by two-sided Student’s t-test.


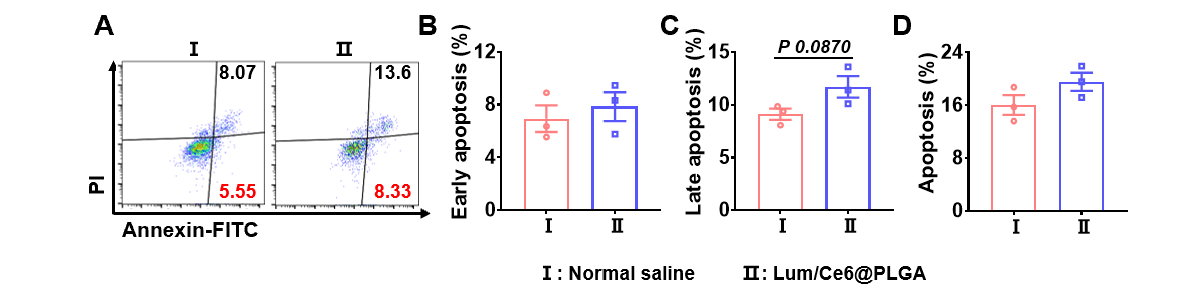


Figure S16. The representative flow cytometry graphs statistical data (A) and the percentages (B-D) of apoptotic neutrophils at 12h after treatment with Lum/Ce6@PLGA nanoparticles.


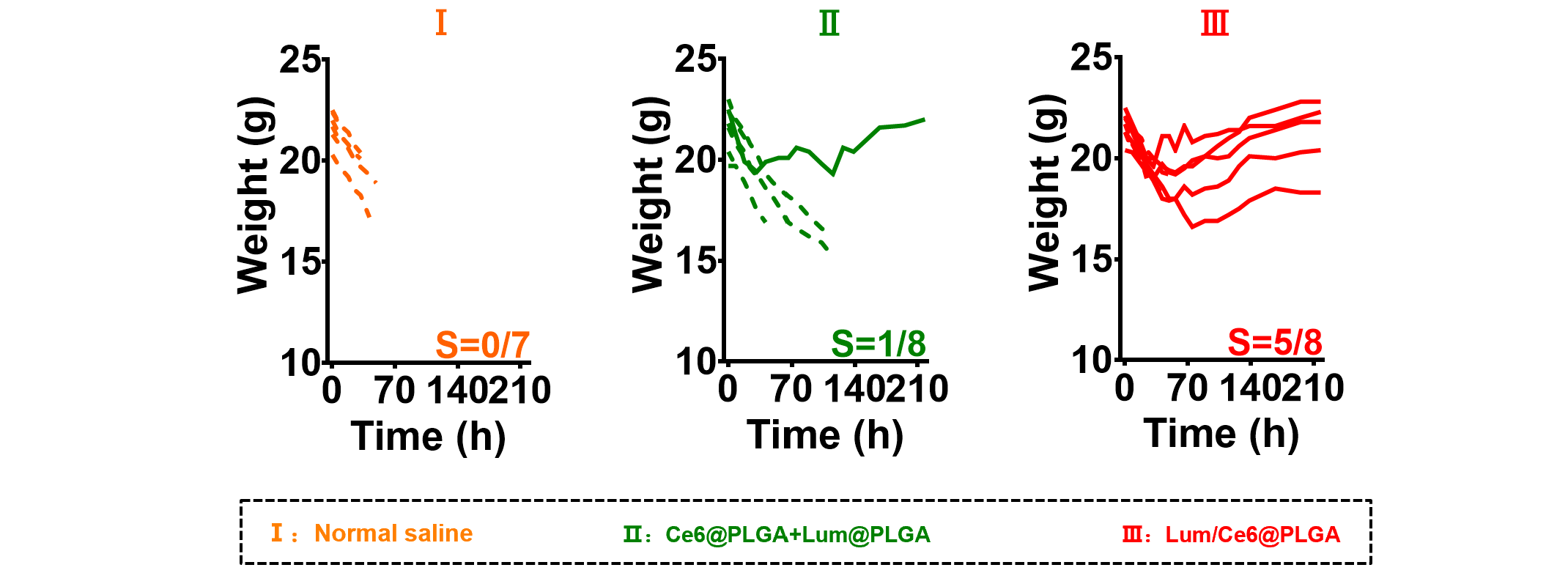


Figure S17. The individual body weights of the peritonitis mice after different treatments.


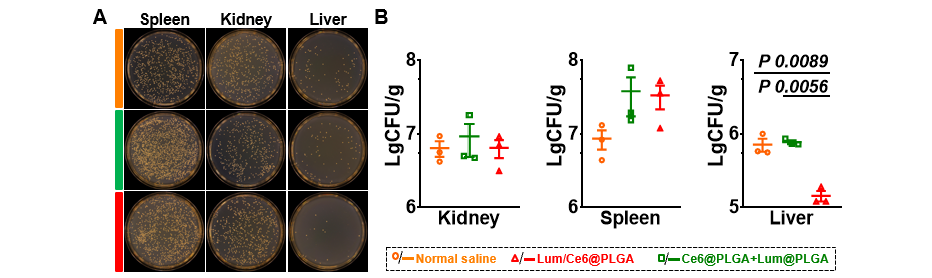


Figure S18. (A&B) Representative images (A) and the statistic data (B) of *S. aureus* colonization in major organs of mice at 12 hours after different treatments as indicated.


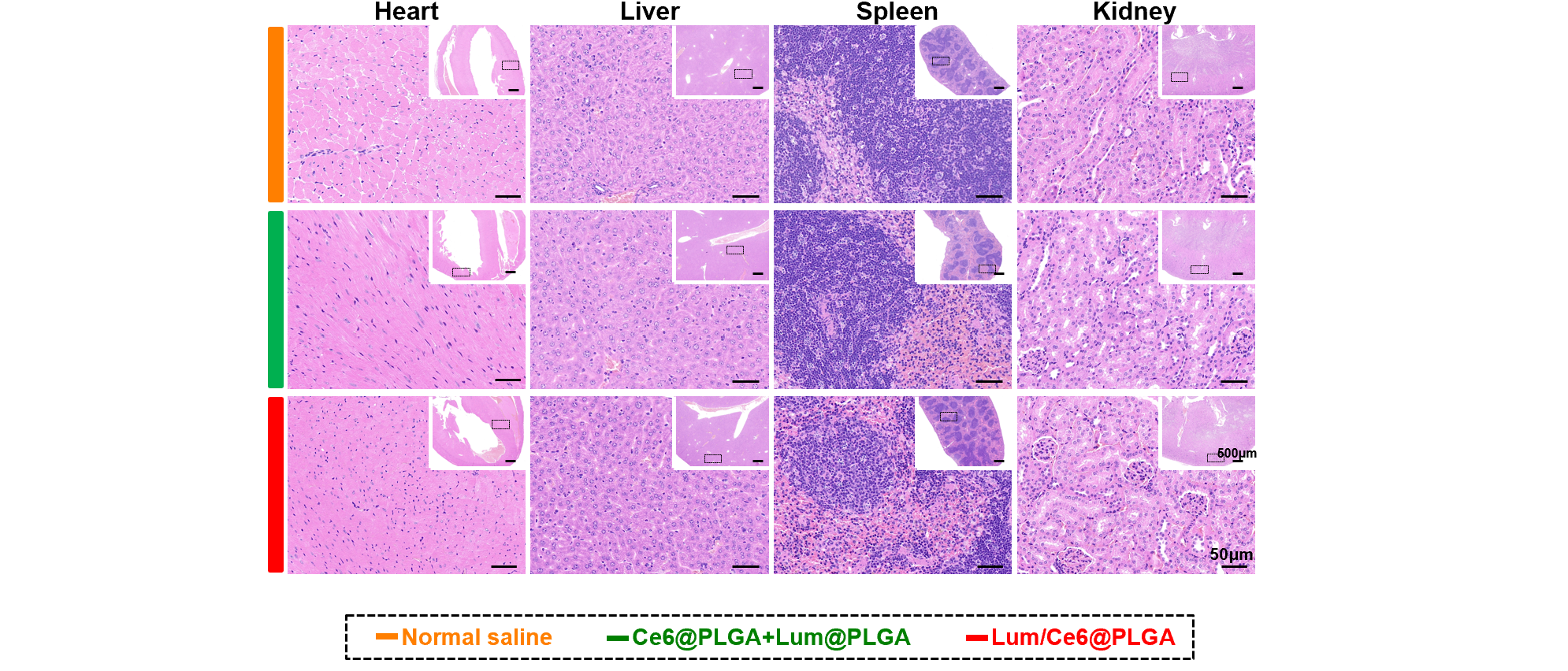


Figure S19. The H&E staining images of major organs collected from mice at 12 hours after different treatments.


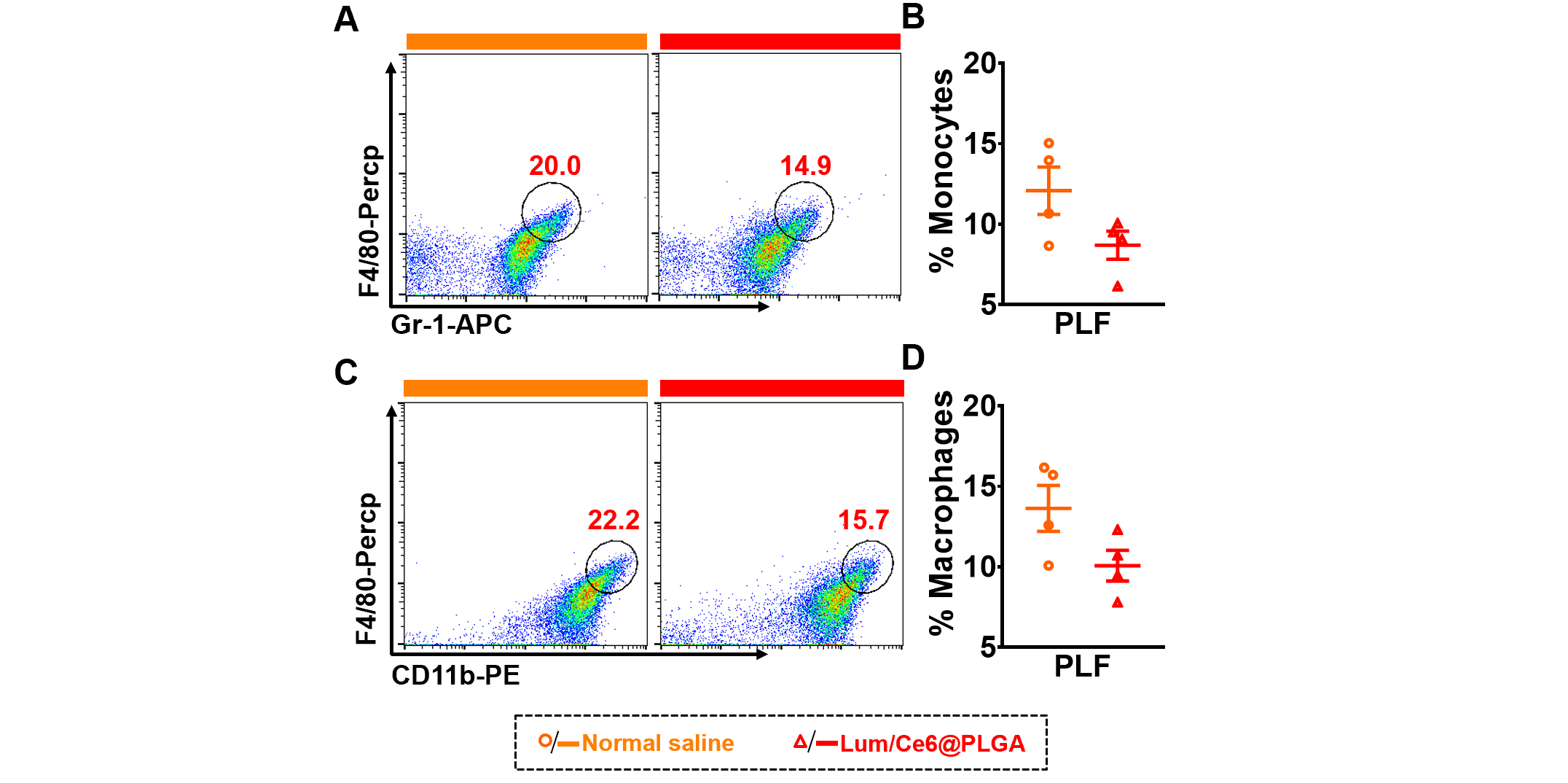


Figure S20. (A-D) Representative flow cytometry graphs and statistical data of the proportions of monocytes (A&B) and macrophages (C&D) in the PLF at 12 hours after different treatments.


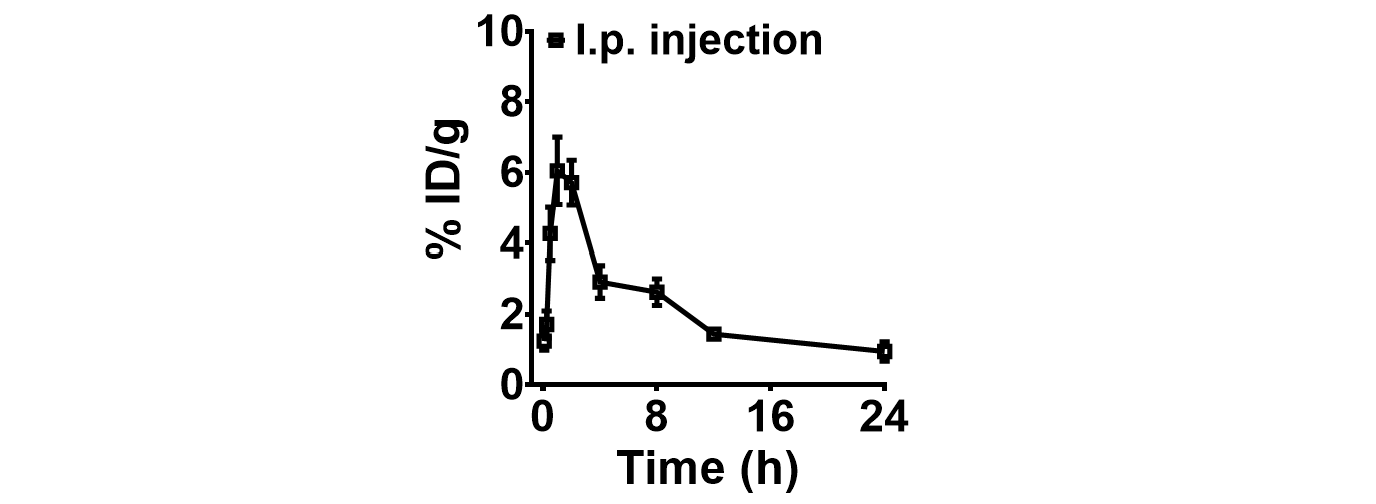


Figure S21. Blood circulation of Lum/Ce6@PLGA nanoparticles post i.p. injection.

*
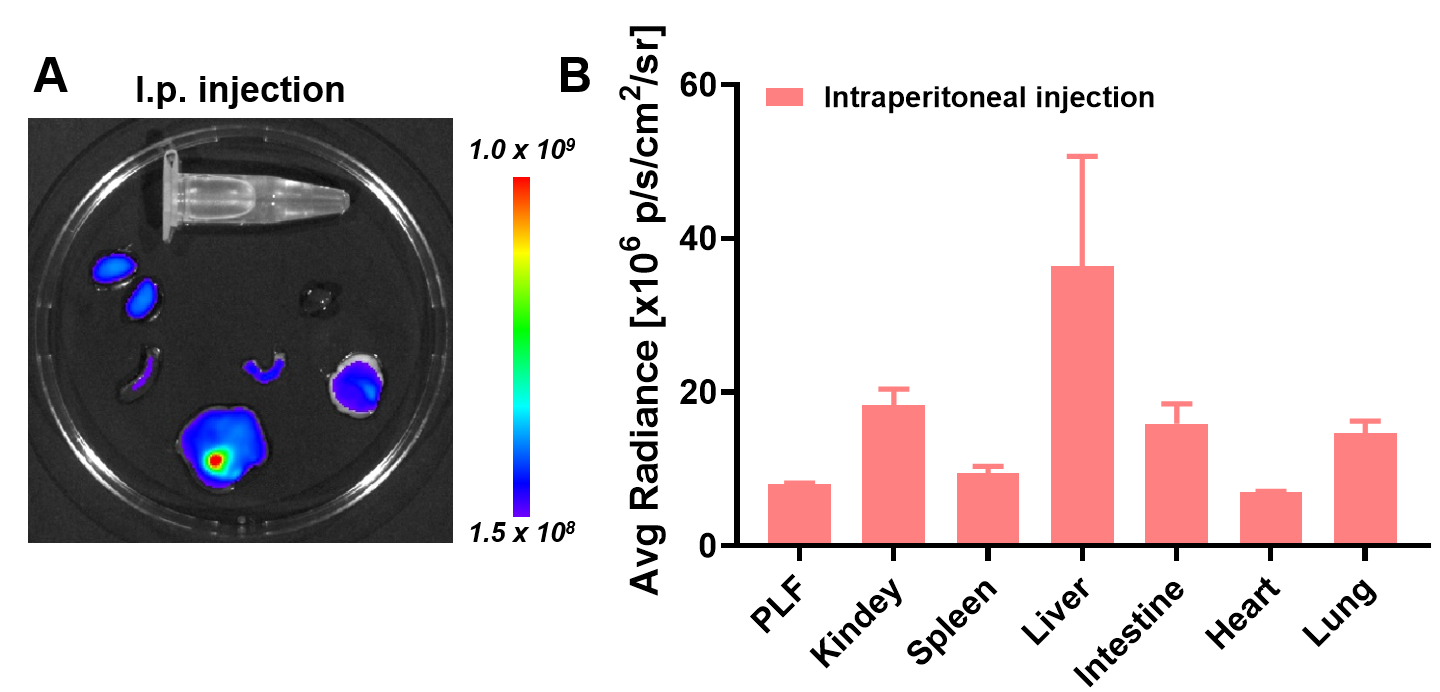
*

Figure S22. *In vivo* fluorescence imaging (A) and quantification (B) of Ce6 in PLF and major organs collected from the *S.aureus*-induced peritonitis mice at 24 h post intraperitoneal injection.


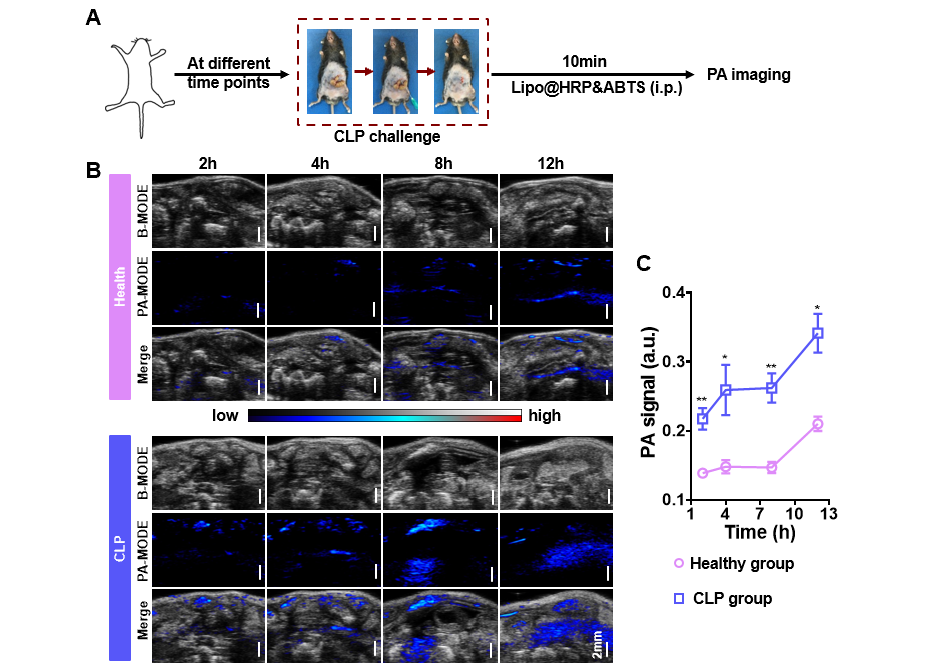


Figure S23. (A) A schematic illustration of photoacoustic imaging of CLP for *in vivo* H_2_O_2_ detection with photoacoustic nanoprobe, Lipo@HRP&ABTS. (B) *In vivo* photoacoustic images of mouse abdomen at different time points after CLP induced sepsis. (C) H_2_O_2_-specifici photoacoustic signals from mouse abdomen of all groups based on PA imaging data in (B). Data are presented as mean ± SEM. Statistical significance was calculated by two-sided Student’s t-test.


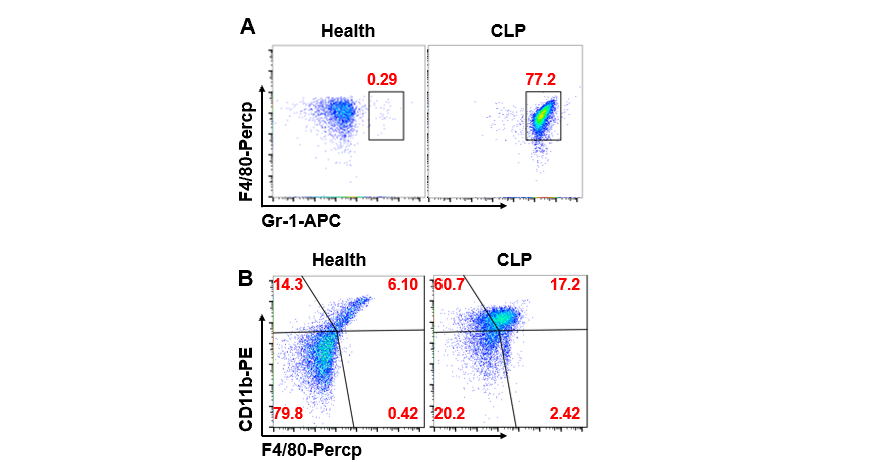


Figure S24. (A&B) Representative flow cytometry graphs of the proportions of monocytes (A) macrophages (B) in PLF collected from the healthy and peritonitis mice at 8 hours after *S. aureus* infection. Data are presented as mean ± SEM. Statistical significance was calculated by two-sided Student’s t-test.


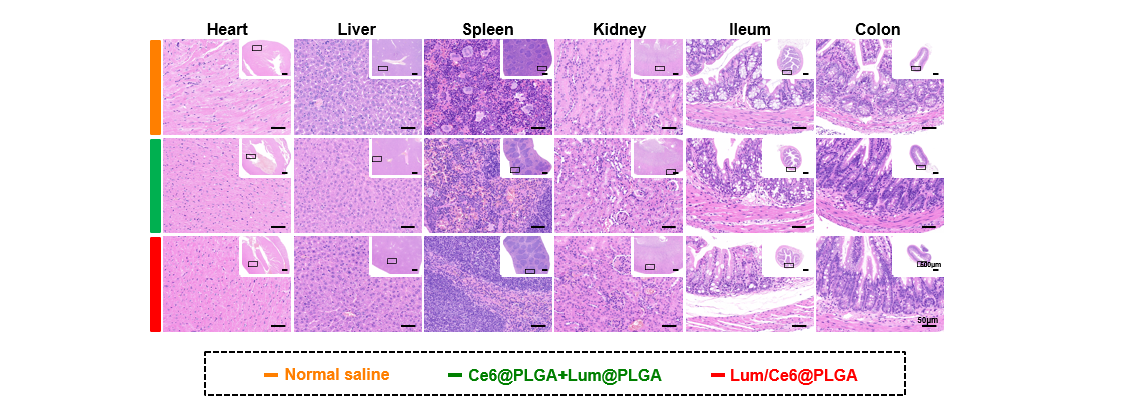


Figure S25. The H&E staining images of major organs collected from mice at 12 hours after different treatments.


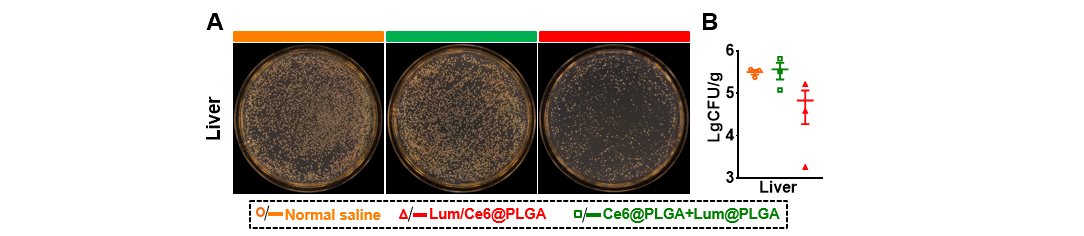


Figure S26. (A&B) Representative images (A) and the statistic data (B) of bacteria colonization in liver of mice at 12 hours after different treatments as indicated. Data are presented as mean ± SEM.


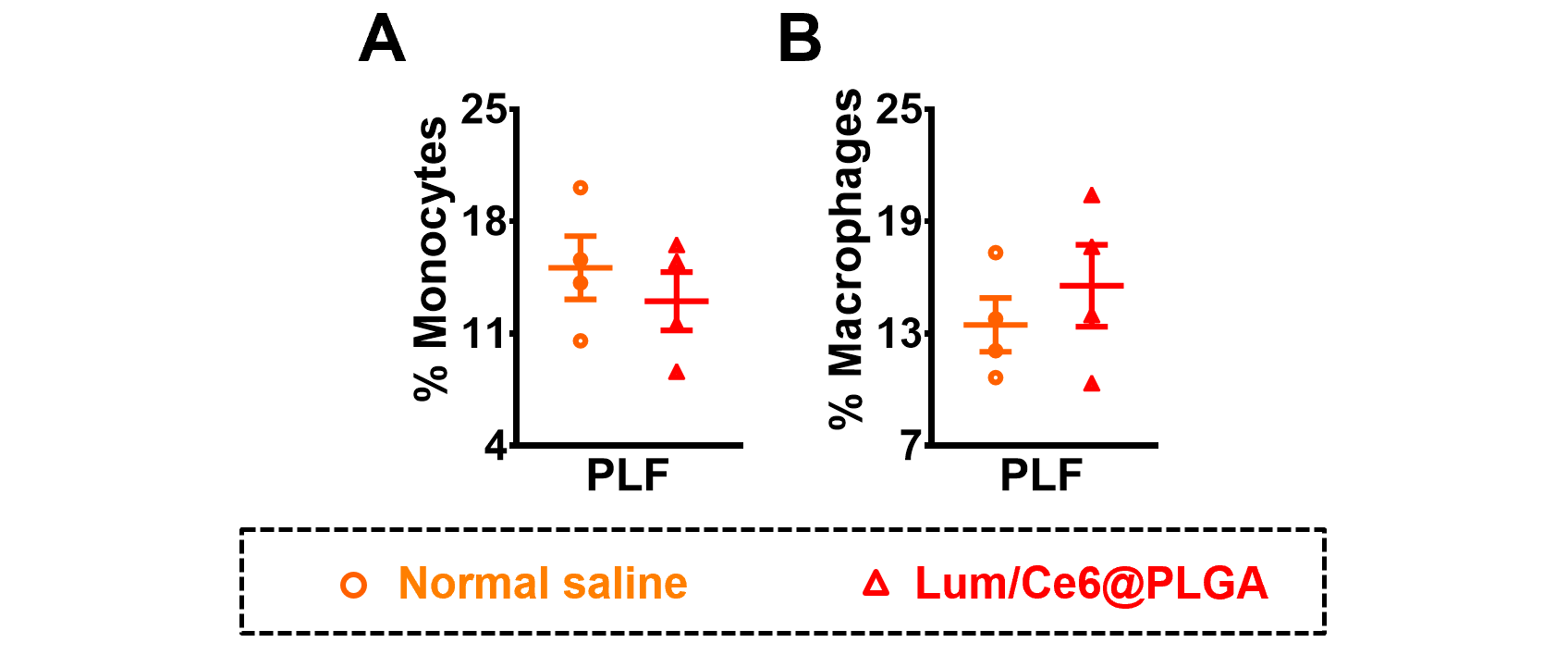


Figure S27. (A&B) The statistical data of the proportions of monocytes (A) and macrophages (B) in the PLF collected at 12 hours after different treatments. Data are presented as mean ± SEM.


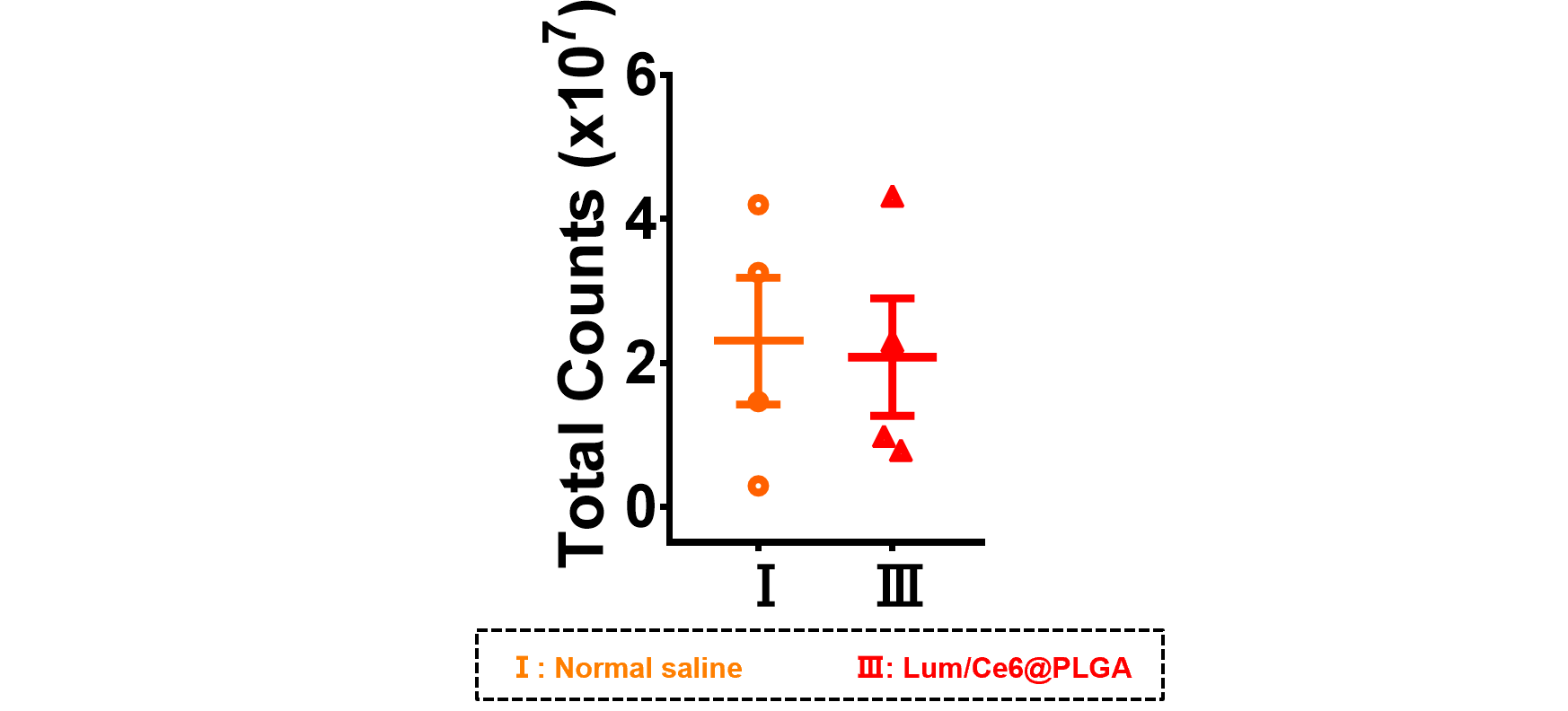


Figure S28. The total number of cells in PLF after different treatments. Data are presented as mean ± SEM.


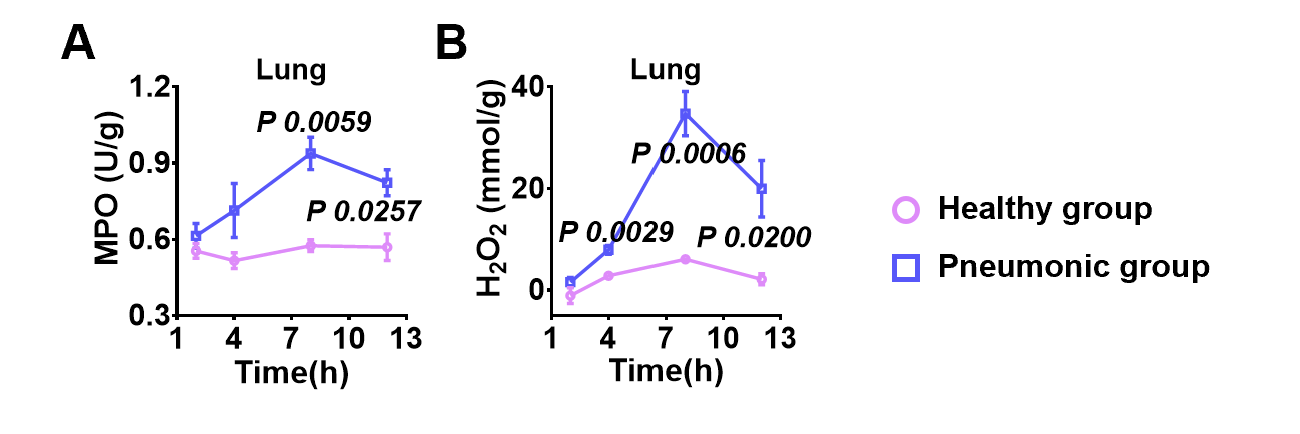


Figure S29. The activities of MPO (A) and concentrations of H_2_O_2_ in lung tissues at different time points in the pneumonia mice.


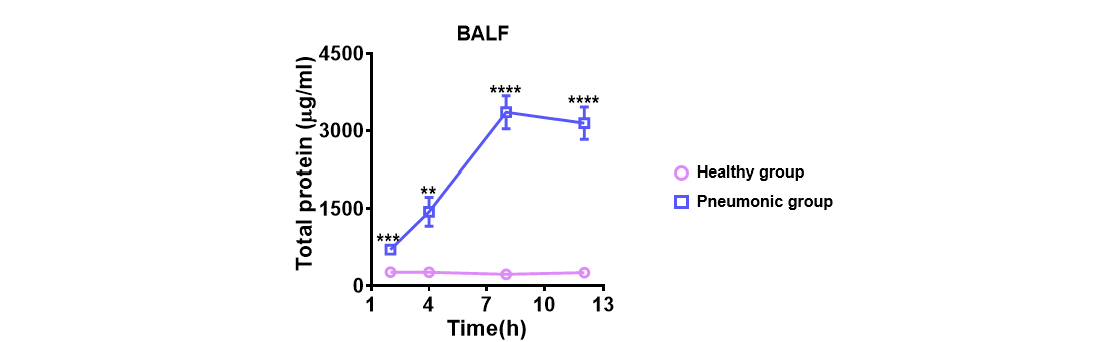


Figure S30. The concentrations of total protein in BALF at different time points. Data are presented as mean ± SEM. Statistical significance was calculated by two-sided Student’s t-test.


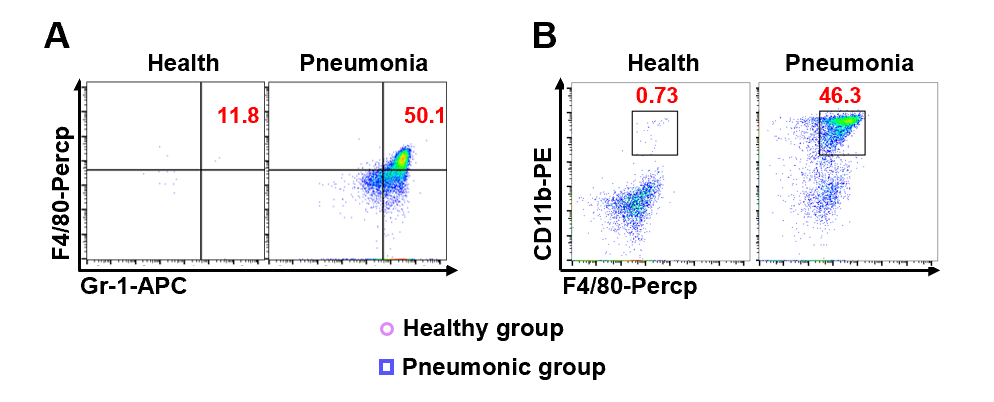


Figure S31. Representative flow cytometry graphs of monocytes (A) and macrophages (B) in BALF collected from healthy and pneumonia mice at 8 hours after infection.


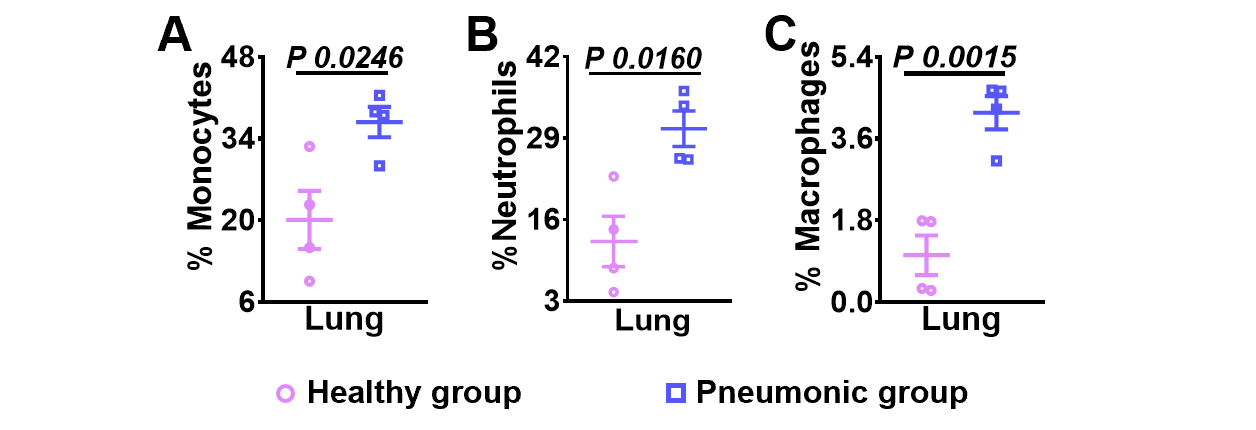


Figure S32. (A-C) The statistical data of the proportions of monocytes (A), neutrophils (B) and macrophages (C) in lung tissues collected from healthy and pneumonic mice at 8 hours after *S. aureus* infection. Data are presented as mean ± SEM. Statistical significance was calculated by two-sided Student’s t-test.


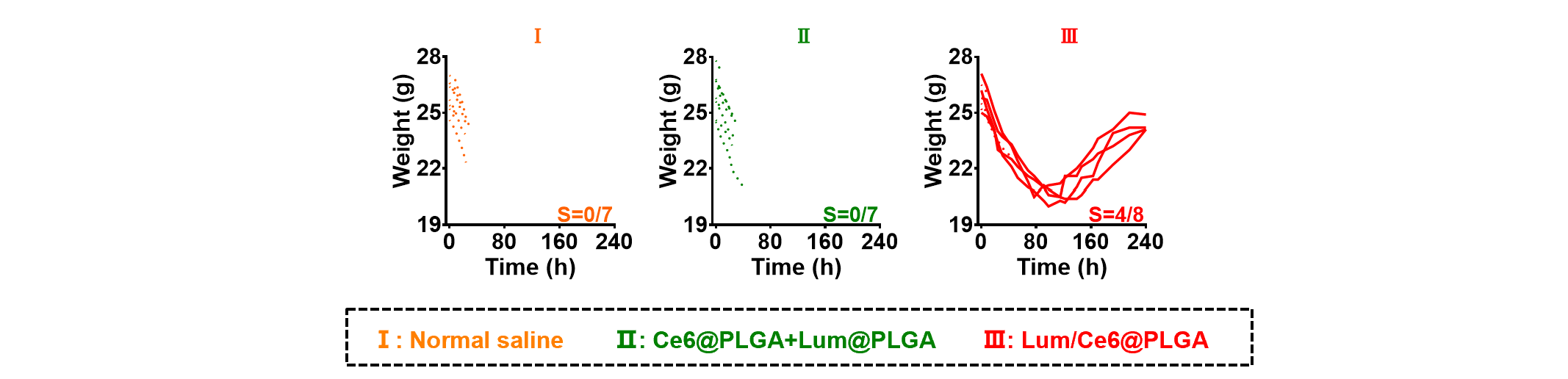


Figure S33. The individual body weights of the peritonitis mice after different treatments.


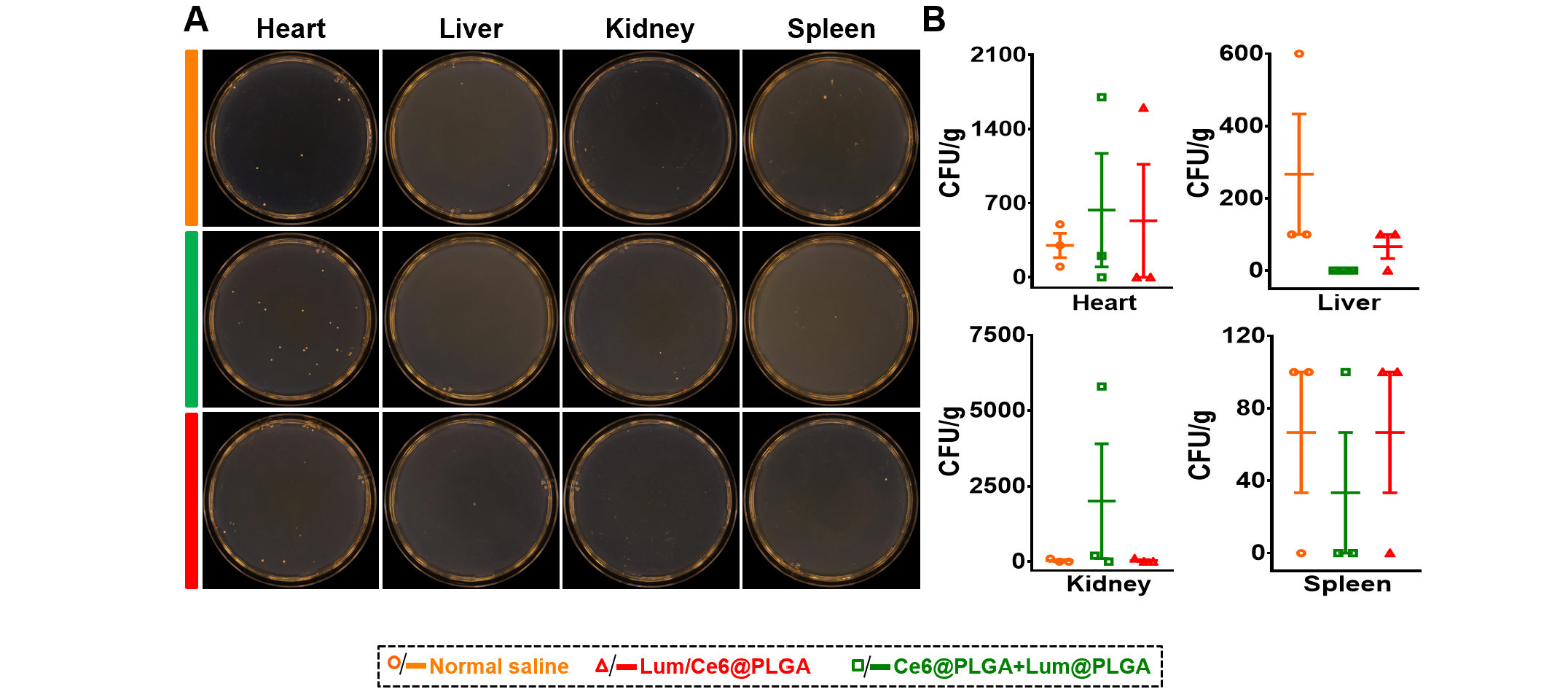


Figure S34. (A&B) Representative images (A) and the statistic data (B) of *S. aureus* colonization in major organs of the mice at 12 hours after different treatments as indicated. Data are presented as mean ± SEM.


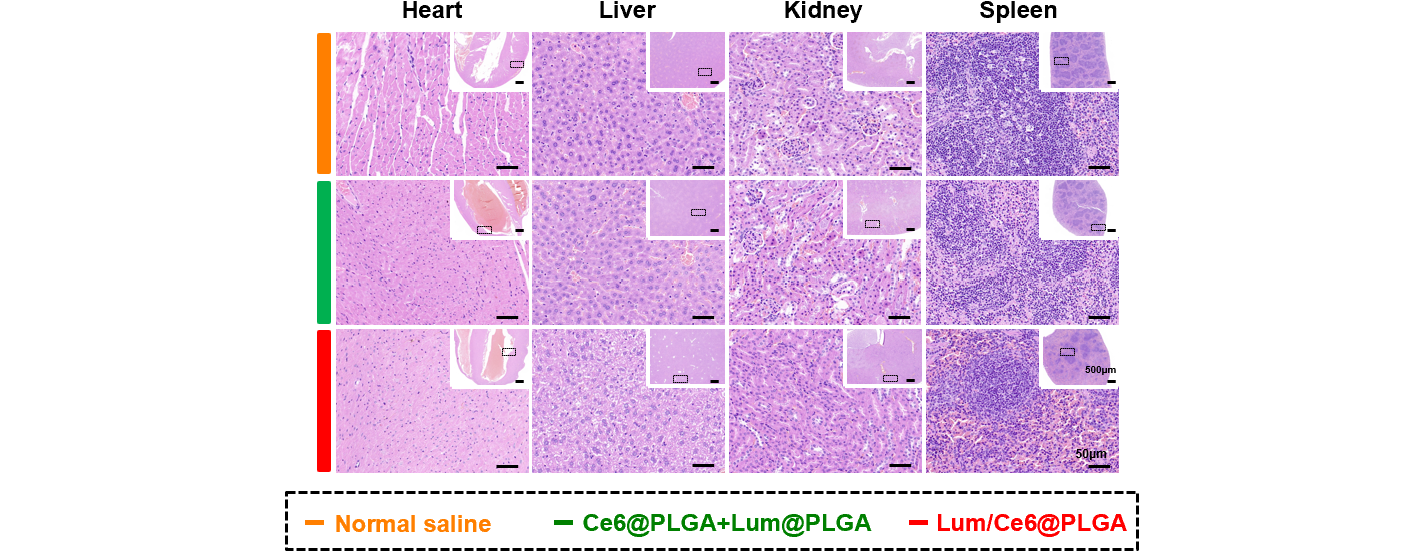


Figure S35. The H&E staining images of major organs collected from mice at 12 hours after different treatments.


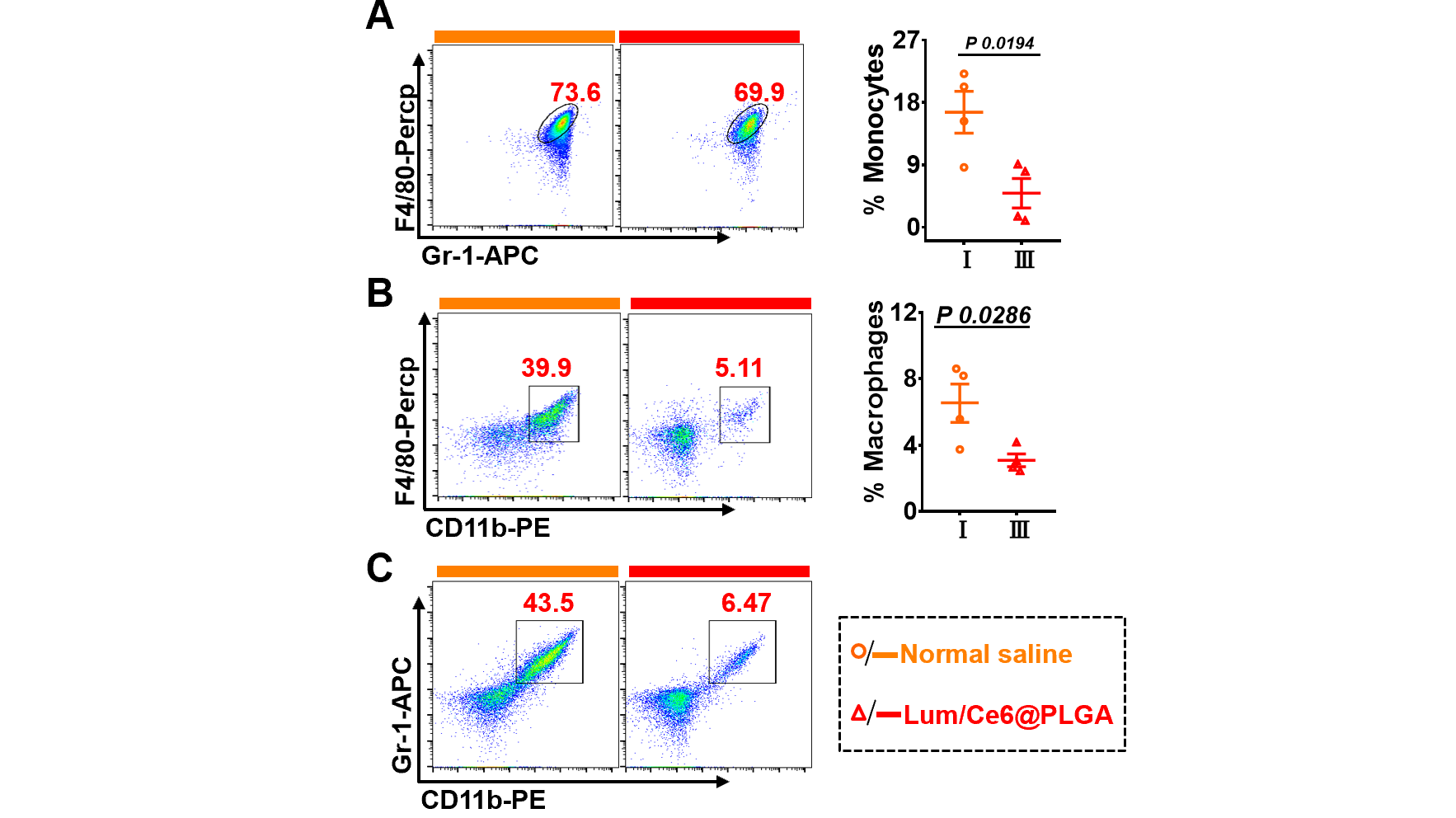


Figure S36. Representative flow cytometry graphs of monocytes (A), macrophages (B) and neutrophils (C) in BALF collected from the pneumonic mice at 12 hours after different treatments.


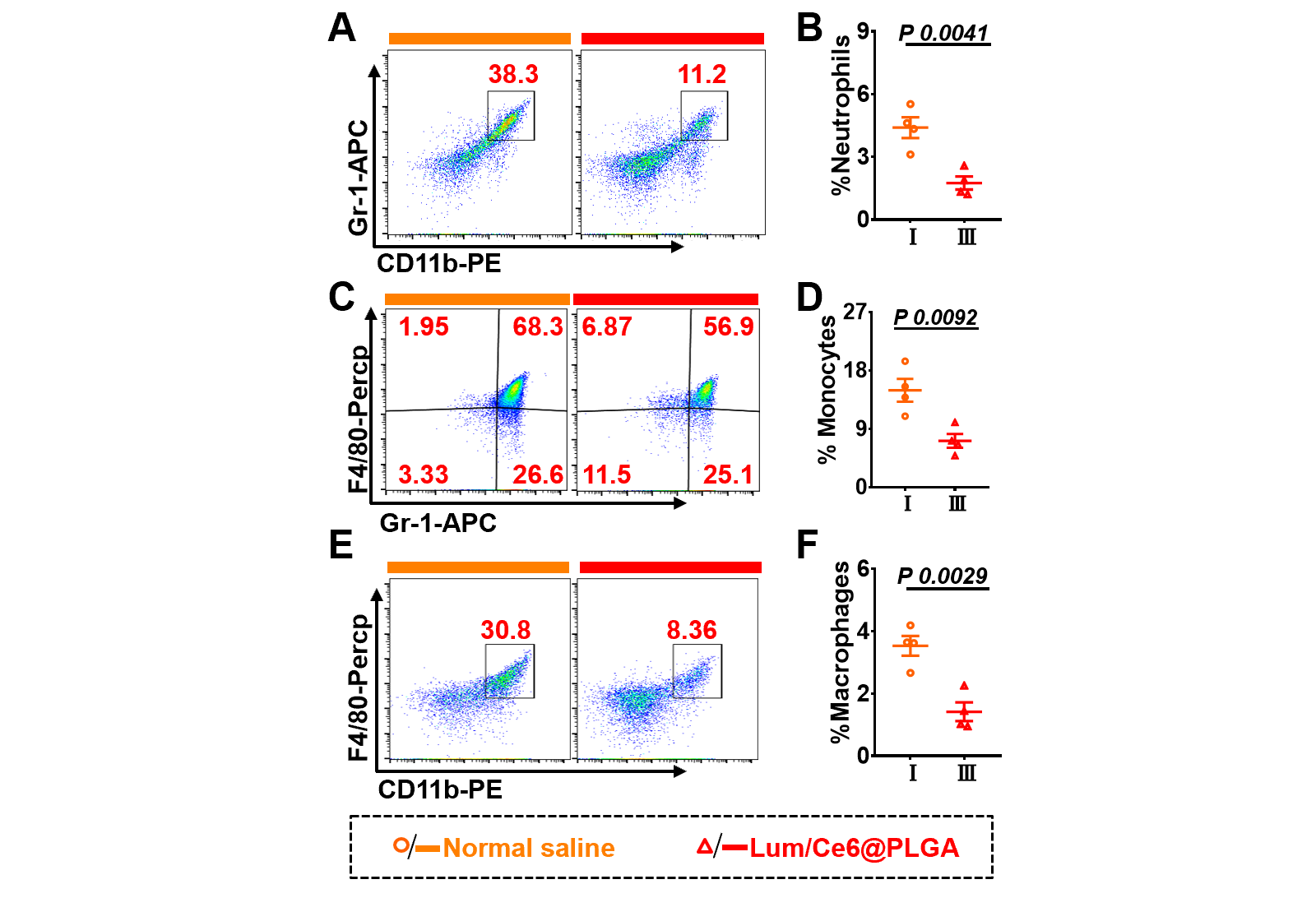


Figure S37.Representative flow cytometry graphs and statistical data of the proportions of neutrophils (A&B), monocytes (B&C) and macrophages (E&F) in lung tissues at 12 hours after different treatments. Data are presented as mean ± SEM. Statistical significance was calculated by one-way ANOVA with Tukey’s post hoc test.


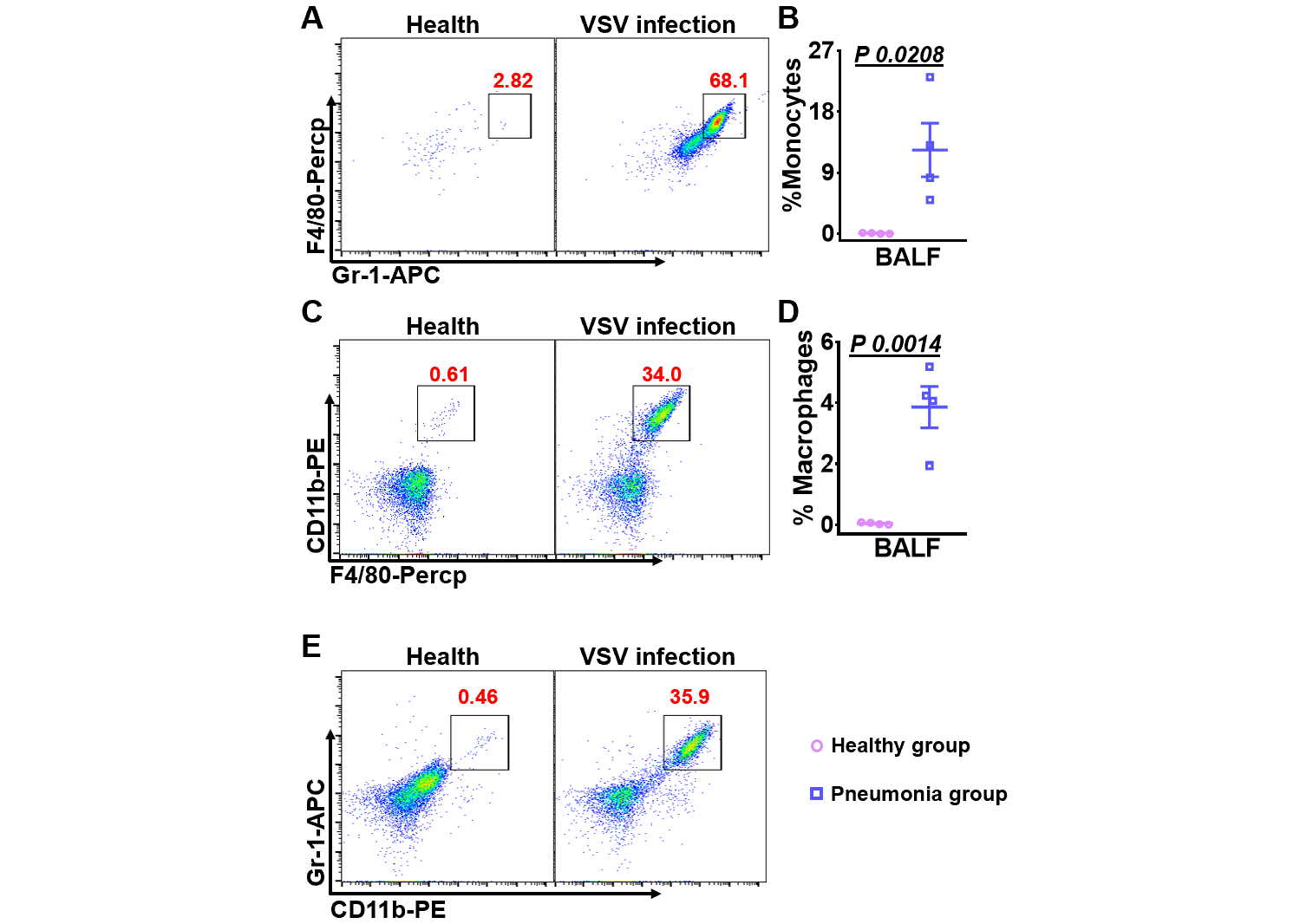


Figure S38. Representative flow cytometry graphs and statistical data of the proportions of monocytes (A&B), macrophages (C&D) and neutrophils (E) in BALF at 8 hours after VSV infection. Data are presented as mean ± SEM. Statistical significance was calculated by two-sided Student’s t-test.


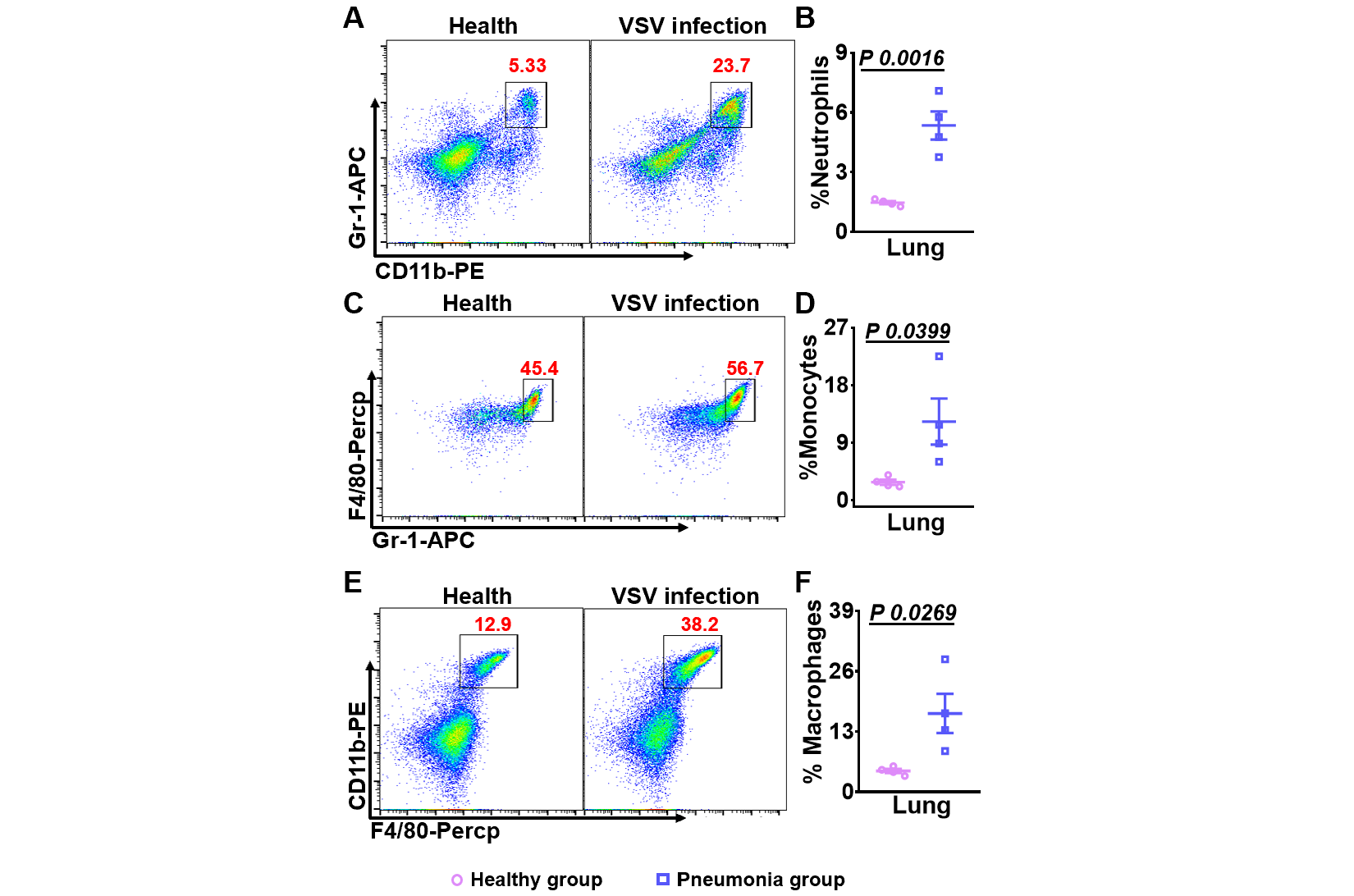


Figure S39. Representative flow cytometry graphs and statistical data of the proportions of neutrophils (A&B), monocytes (C&D) and macrophages (E&F) in lung tissues at 8 hours after VSV infection. Data are presented as mean ± SEM. Statistical significance was calculated by two-sided Student’s t-test.


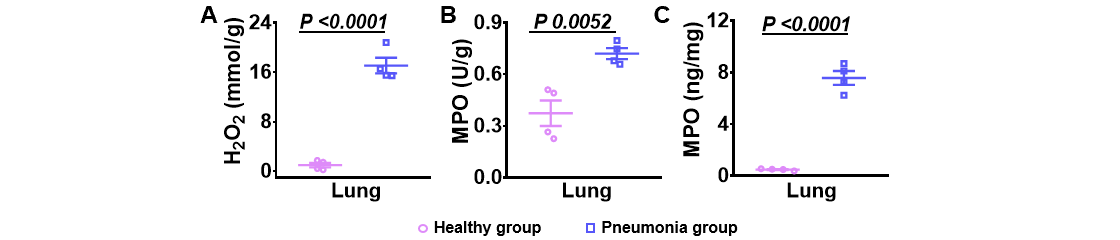


Figure S40. (A) The concentration of H_2_O_2_ in lung tissues of pneumonia mice at 8 h post-infection. (B&C) The activity (B) and concentration (C) of MPO in lung tissues at 8 h post-infection. Data are presented as mean ± SEM. Statistical significance was calculated by two-sided Student’s t-test.


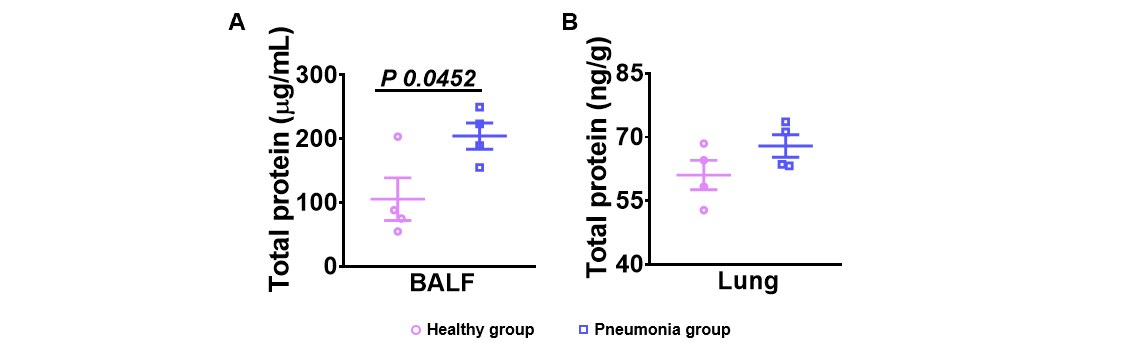


Figure S41. (A&B) The concentrations of total protein in BALF (A) and lung tissues (B) of pneumonia mice at 8 h post-infection. Data are presented as mean ± SEM. Statistical significance was calculated by two-sided Student’s t-test.


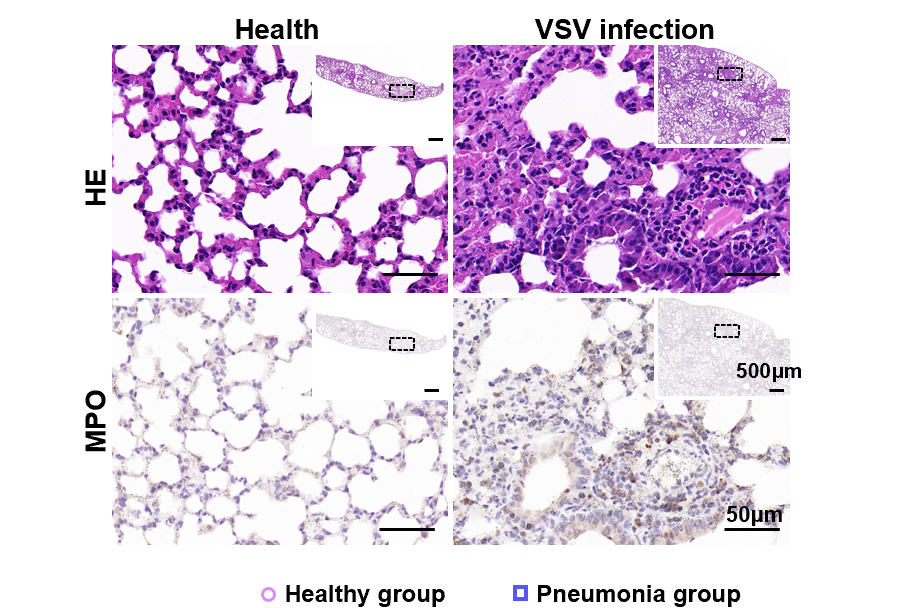


Figure S42. The H&E staining images and MPO-specific IHC staining images of lung tissues collected at 8 hours after VSV infection.


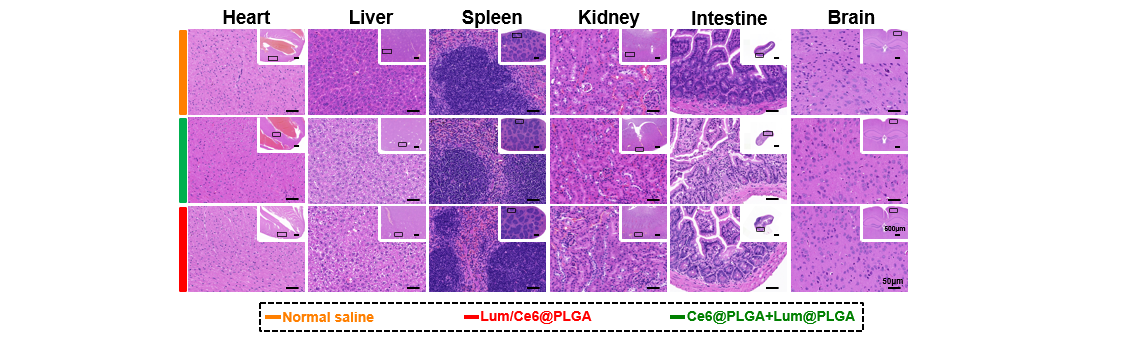


Figure S43. The H&E staining images of major organs of mice collected after different treatments.


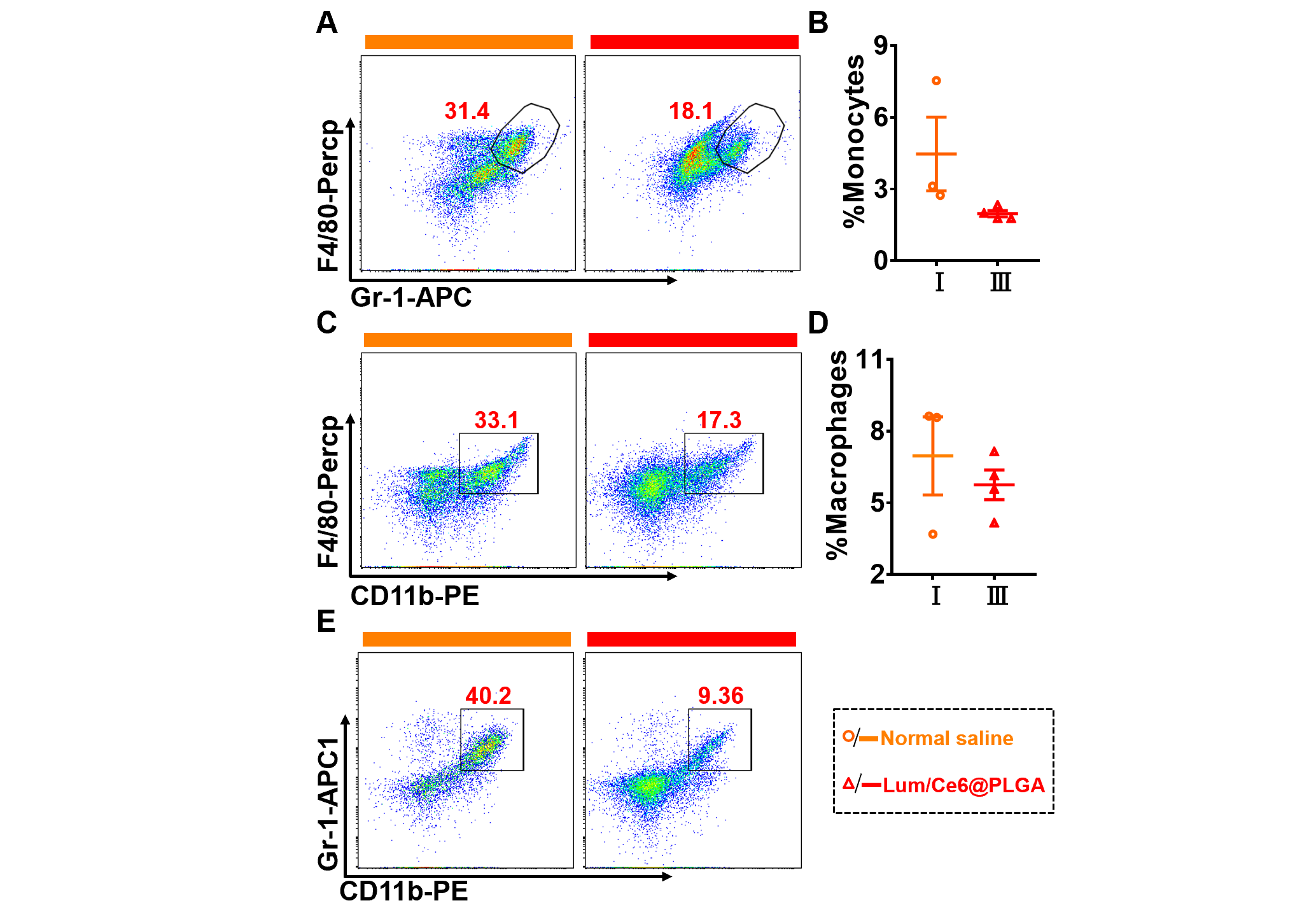


Figure S44. Representative flow cytometry graphs and statistical data of the proportions of monocytes (A&B), macrophages (C&D) and neutrophils (E) in BALF after different treatments. Data are presented as mean ± SEM.


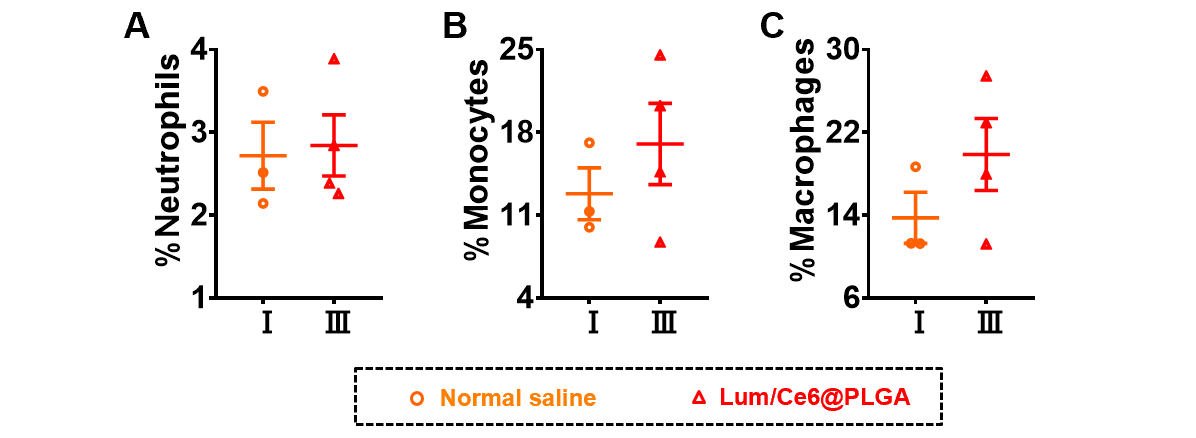


Figure S45. (A-C) The statistical data of the proportions of neutrophils (A), monocytes (B) and macrophages (C) in lung tissues after different treatments. Data are presented as mean ± SEM.


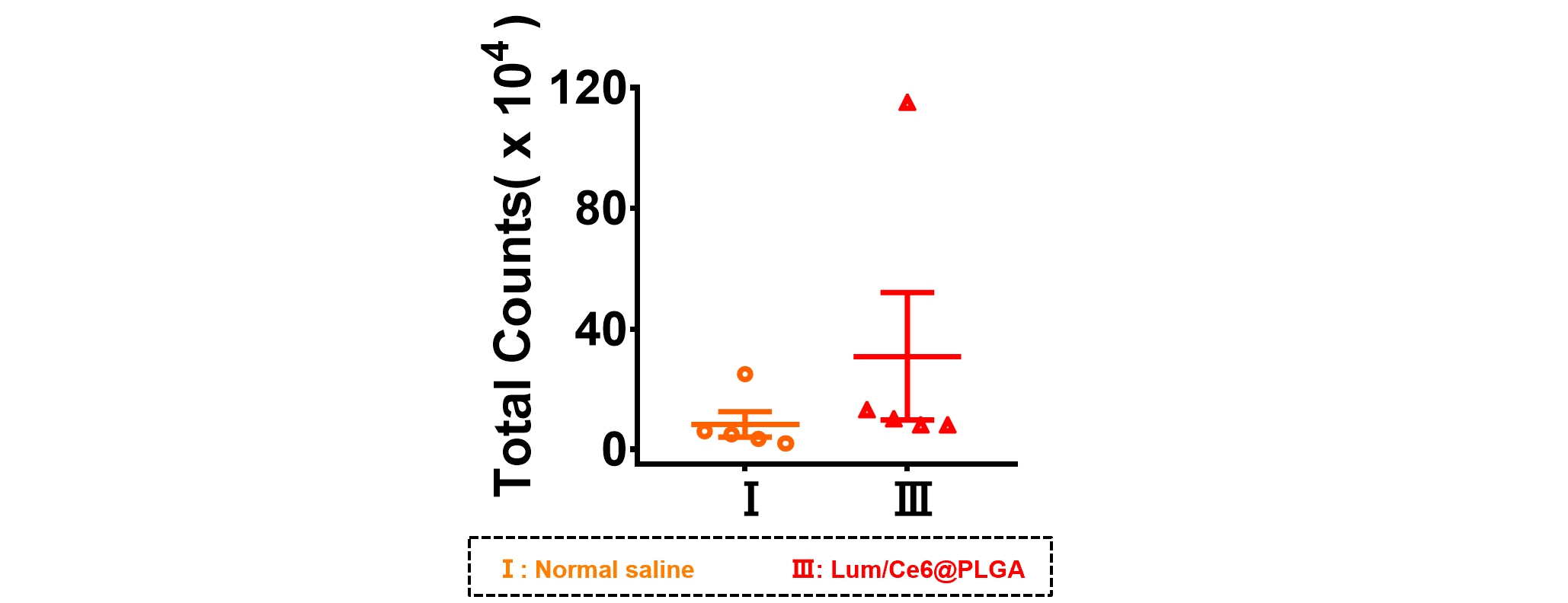


Figure S46. The total cell counts in BALF after different treatments. Data are presented as mean ± SEM.


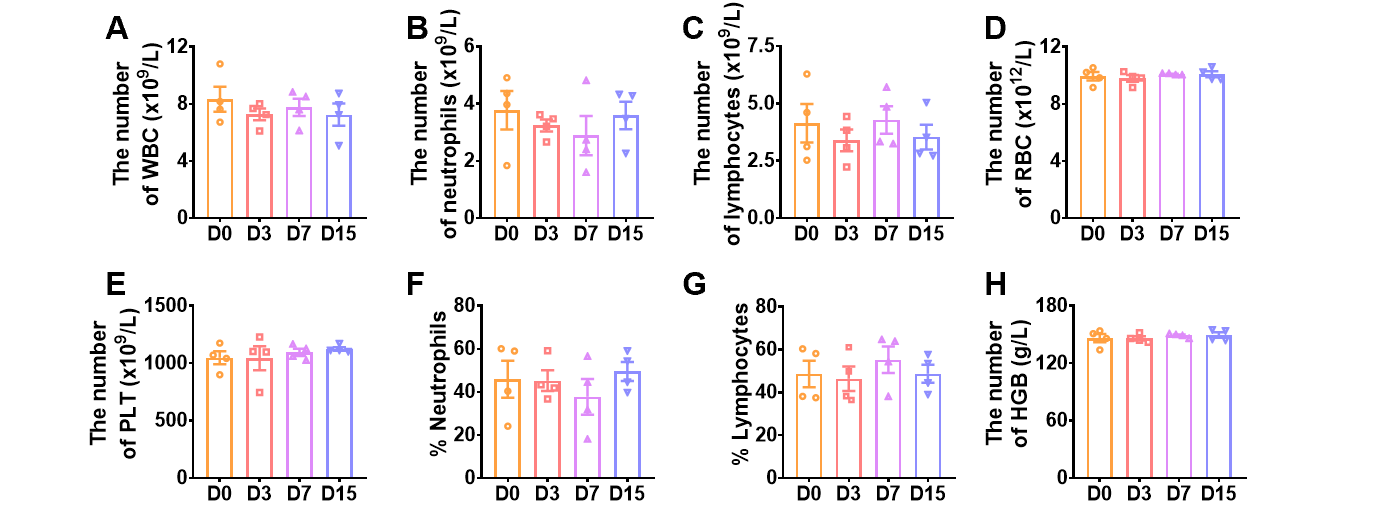


Figure S47. The complete blood panel analysis of the mice after i.p. injection of Lum/Ce6@PLGA nanoparticles (at the Ce6 dosage of 80 μg) collected at 0, 3, 7 and 15 days p.i. (white blood cells (WBC), red blood cells (RBC), platelet (PLT) and hemoglobin (HGB)).


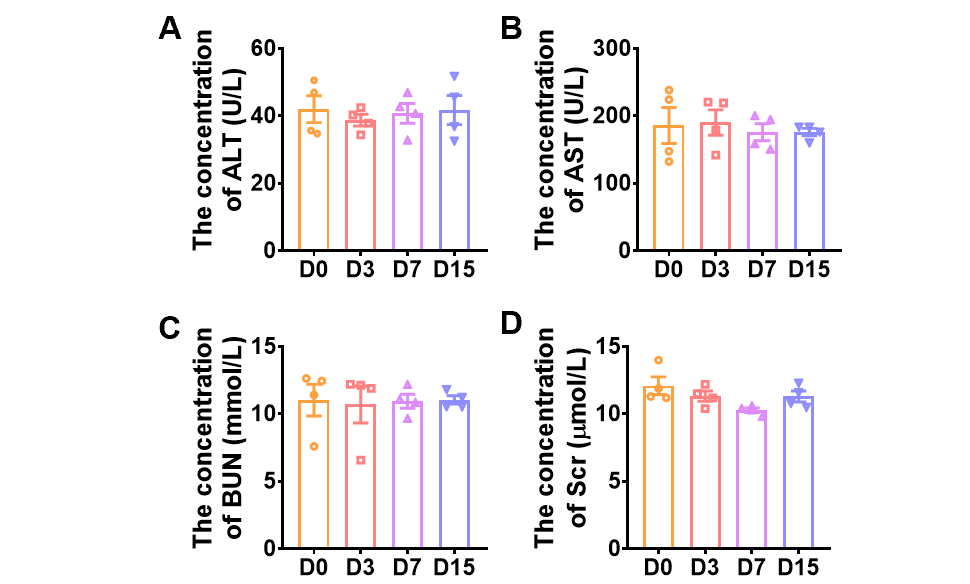


Figure S48. The blood biochemistry panel analysis of the mice after i.p. injection of Lum/Ce6@PLGA nanoparticles (at the Ce6 dosage of 80 μg) collected at 0, 3, 7 and 15 days p.i. (alanine aminotransferase (ALT), aspartate aminotransferase (AST), blood urea nitrogen (BUN) and serum creatinine (Scr)).


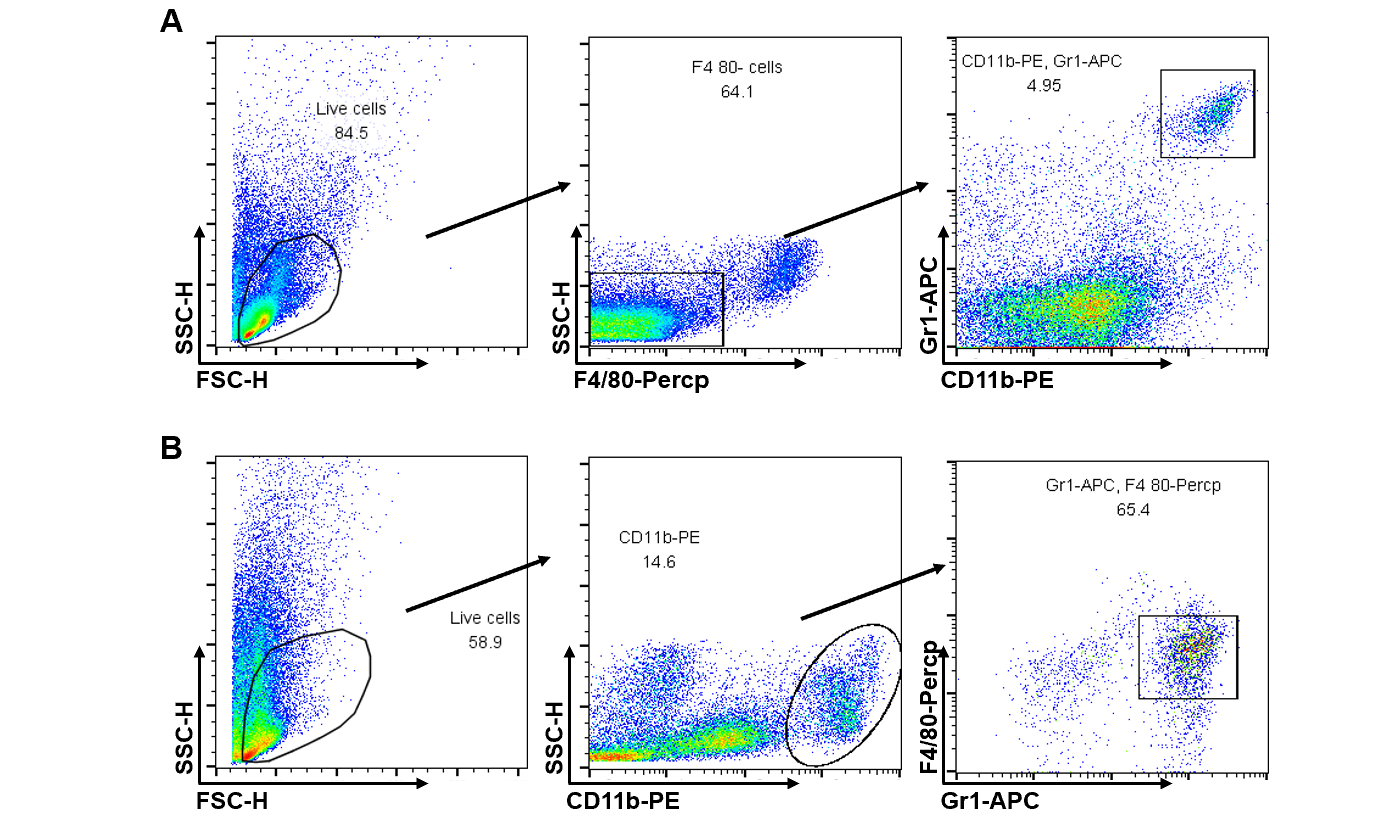


Figure S49. Gating strategy to determine the percentage of neutrophils (A) and monocytes (B).

**References**

1. Chen, Q. et al. H(2)O(2)-responsive liposomal nanoprobe for photoacoustic inflammation imaging and tumor theranostics via in vivo chromogenic assay. *Proc. Natl. Acad. Sci. U. S. A.* **114**, 5343-5348 (2017).
